# Device-measured sedentary time and intensity-specific physical activity in relation to all-cause and cardiovascular disease mortality: the UK Biobank cohort study

**DOI:** 10.1186/s12966-024-01615-5

**Published:** 2024-07-03

**Authors:** Leandro F. M. Rezende, Matthew Ahmadi, Gerson Ferrari, Borja del Pozo Cruz, I-Min Lee, Ulf Ekelund, Emmanuel Stamatakis

**Affiliations:** 1 Department of Preventive Medicine, Escola Paulista de Medicina, Universidade Federal de São Paulo, São Paulo, Brazil; 2 Faculty of Health Sciences, Universidad Autónoma de Chile, Providencia, 7500912 Chile; 3 Mackenzie Wearables Research Hub, Charles Perkins Centre, The University of Sydney, Sydney, Australia; 4 School of Health Sciences, Faculty of Medicine and Health, The University of Sydney, Sydney, Australia; 5 Escuela de Ciencias de la Actividad Física, el Deporte y la Salud, Universidad de Santiago de Chile (USACH), Santiago, Chile; 6 Department of Sport Sciences and Clinical Biomechanics, Faculty of Health Sciences, University of Southern Denmark, Campusvej 55, Odense, 5230 Denmark; 7 Faculty of Education, University of Cádiz, Cádiz, Spain; 8 Biomedical Research and Innovation Institute of Cádiz (INiBICA) Research Unit, University of Cádiz, Cádiz, Spain; 9 Division of Preventive Medicine, Brigham & Women’s Hospital, Harvard Medical School, Boston, USA; 10 Department of Epidemiology, Harvard T.H. Chan School of Public Health, Boston, MA USA; 11 Department of Sport Medicine, Norwegian School of Sports Sciences, Oslo, Norway; 12 Department of Chronic Diseases, Norwegian Institute of Public Health, Oslo, Norway

**Keywords:** Physical activity, Sedentary behaviour, Accelerometer, Cardiovascular disease mortality, All-cause mortality

## Abstract

**Background and aims:**

Understanding the amounts of intensity-specific movement needed to attenuate the association between sedentary time and mortality may help to inform personalized prescription and behavioral counselling. Herein, we examined the joint associations of sedentary time and intensity-specific physical activity with all-cause and cardiovascular disease (CVD) mortality.

**Methods:**

Prospective cohort study including 73,729 adults from the UK Biobank who wore an Axivity AX3 accelerometer on their dominant wrist for at least 3 days, being one a weekend day, between June 2013 and December 2015. We considered the median tertile values of sedentary time and physical activity in each intensity band to determine the amount of physical activity needed to attenuate the association between sedentary time and mortality.

**Results:**

During a median of 6.9 years of follow-up (628,807 person-years), we documented 1521 deaths, including 388 from CVD. Physical activity of any intensity attenuated the detrimental association of sedentary time with mortality. Overall, at least a median of 6 min/day of vigorous physical activity, 30 min/day of MVPA, 64 min/day of moderate physical activity, or 163 min/day of light physical activity (mutually-adjusted for other intensities) attenuated the association between sedentary time and mortality. High sedentary time was associated with higher risk of CVD mortality only among participants with low MVPA (HR 1.96; 95% CI 1.23 to 3.14).

**Conclusions:**

Different amounts of each physical activity intensity may attenuate the association between high sedentary time and mortality.

**Supplementary Information:**

The online version contains supplementary material available at 10.1186/s12966-024-01615-5.

## Introduction

The World Health Organization (WHO) recognizes that high amounts of sedentary behavior are associated with all-cause, cardiovascular disease (CVD), and cancer mortality and incidence of CVD, cancer and type 2 diabetes. Moderate certainty of evidence suggests that adults should limit the amount of time spent in sedentary activities; and that replacing sedentary time with physical activity of any intensity provides health benefits [1]. In fact, epidemiological studies suggest that moderate to vigorous physical activity (MVPA) attenuates the association between high sedentary behavior/time and increased risk of mortality [2–4]. However, these findings were mostly based on self-reported data, which reflect behavior and incorporate measurement error of actual movement performed. In addition, previous self-report studies did not cover the amount of intensity-specific movement, which may be useful to inform personalized prescription and behavioral counselling. Of note, VPA generally shows a greater magnitude of association with lower risk of mortality compared to MPA [5–7].

Enabled by advances in research-grade technology, recent studies of wearable movement sensors (accelerometers) have highlighted the health relevance of physical activity intensity bands, including light (LPA) [8] and VPA. [5, 6] Large pooled harmonized meta-analyses of waking times accelerometry and cohort studies of self-reported data [2–4] have shown that the associations of sedentary time and risk of all-cause [9–15] and cardiovascular disease (CVD) [9, 16, 17] mortality are dependent on a MVPA level [2–4]. To our knowledge, no device-based study has examined the joint association of sedentary time and different physical activity intensities with mortality risk in general adult population [18]. The joint association approach allows to create a classification that comprised mutually exclusive combinations of sedentary time and each intensity-specific physical activity exposure, mutually accounting for other physical activity intensities and device-estimated sleep duration. Joint analyses, although distinctively different from compositional data analysis (CODA), [19, 20] also take into account most components of 24-hour physical behaviour.

Capitalizing on the largest 24-h accelerometry data resource available and a validated machine learning generated movement classifier, [5, 6, 21] we examined the joint associations of sedentary time and intensity-specific physical activity with all-cause and CVD mortality in UK adults. We also aimed to determine the daily amount of movement performed at MVPA, MPA, VPA and LPA intensities needed to attenuate the association between sedentary time and mortality.

## Methods

### Study design and sample

The UK Biobank enrolled over 500,000 UK adults from 22 centres across the United Kingdom between 2006 and 2010. The study protocol was approved by the institutional review boards of the National Health Service and the National Research Ethics Service (reference 11/NW/0382), and all participants provided consent for access to their national health records. In this study, we included participants with at least 3 days (with at least one of the days being a weekend day) of valid accelerometer data between June 2013 and December 2015. Using self-report, hospital admission, and cancer registry records, we excluded participants diagnosed with CVD, cancer or respiratory diseases (*n* = 17,242) prior to the accelerometry baseline. Lastly, we excluded deaths within the first two years of follow-up (*n* = 488) to reduce the possibility of reverse causation bias, [22–24] producing a final analytical sample of 73,729 participants.

### Assessment of sedentary time and physical activity intensity

A total of 103,684 participants were mailed and wore an Axivity AX3 accelerometer (Newcastle upon Tyne, UK) on their dominant wrist for 24-hours/day for 7 days to measure movement performed at different intensities. Prior to being mailed, the AX3 accelerometers were initialized to collect data with a sampling frequency of 100 Hz and a dynamic range between ± 8 g. Participants returned the devices by mail and the data were calibrated and non-wear periods were identified according to standard procedures. [25, 26] Monitoring days were considered valid if wear time was greater than 16 h. Movement intensity was classified with a validated accelerometer-based activity machine learning scheme [27] covering sedentary time, standing utilitarian movements, LPA, MPA, and VPA that has been used in prior cohort studies. [5, 6, 28, 29] The classifier categorized movement in 10 s windows into 1 of 4 movement classes: sedentary, standing utilitarian movements (e.g., ironing a shirt, washing dishes), walking activities (e.g. gardening, active commuting, mopping floors), running/high energetic activities (e.g. active playing with children). These classes were then assigned to 1 of 4 physical activity intensities: sedentary, light, moderate, and vigorous. Walking activities were classified as light (< 100 mg), moderate (≥ 100 mg) and vigorous (≥ 400 mg) intensity. A complete description of the physical activity and sedentary time classifier performance statistics is provided in Supplemental Text 1.

### All-cause and cardiovascular disease mortality ascertainment

Deaths were ascertained through linkage with the National Health Service Digital of England and Wales or the National Health Service Central Register and National Records of Scotland. The International Classification of Diseases 10th version (ICD-10) codes were used to classify the underlying causes of death. All-cause and CVD (I0, I11, I13, I20-I51, I60-I69) mortality were the main outcomes.

### Covariates

Sleep duration (hours/day) was assessed by Axivity AX3 accelerometer. As in previously published analyses, [5, 6, 8, 21] other covariates (listed below) were self-reported at a median of 5.5 years before the accelerometer measures. Information on age, sex, and race and ethnicity (Asian; Black; Mixed; White and Other) were assessed. Participants also reported their highest qualification achieved/educational attainment(National Vocational Qualification (NVQ) or Higher National Diploma (HND) or Higher National Certificate (HNC) or equivalent = 19 years of education; Certificate of Secondary education (CSE) or equivalent = 10 years of education; O levels/General Certificate of Secondary Education (GCSE) or equivalent = 10 years of education; A levels/AS levels or equivalent = 13 years of education; and College or University degree = 20 years of education). Body mass index was calculated by weight in kilogram divided by height in meter squared. Lifestyle risk factors included smoking status (never; former; current smokers), alcohol consumption (never; former; current, below guidelines; current, above guideline; where guideline = < 14 units of alcohol per week and 1 unit = 8 g of pure ethanol)); and fruits and vegetables (servings per day). Health status, family history of CVD and cancer, and current use of diabetic and blood pressure medication were also assessed.

### Statistical analysis

Person-time was calculated from assessment of accelerometer data (June 2013 and December 2015) until the time of death, or the end of the follow up period (September 30, 2021 for England and Wales and October 31, 2021 for Scotland), whichever occurred first. Multivariable Cox proportional hazard regression models were performed to estimate the hazard ratio (HR) and 95% confidence interval (95% CI) for the joint association of sedentary time and physical activity intensities with all-cause and CVD mortality. Tertiles were used to categorize low (3.1 to 10.1 h/day), medium (10.2 to 11.3 h/day) and high (≥ 11.3 h/day) sedentary time. We also used tertiles to categorize MVPA (0 to 21.2 min/day; 21.3 to 40.9 min/day; ≥41 min/day), LPA (0 to 70.2 min/day; 70.3 to 119.2 min/day; ≥119.3 min/day), MPA (0 to 19.1 min/day; 19.2 to 36.8 min/day; ≥36.9 min/day), and VPA (0 to 0.97 min/day; 0.98 to 2.9 min/day; ≥3 min/day).

We determined the amount of physical activity needed to attenuate the association between sedentary time and mortality by using median tertile values of physical activity in each intensity band: MVPA (low: 13.5 min/day; medium: 30.0 min/day; high: 59.3 min/day), LPA (low: 54.1 min/day; medium: 90.4 min/day; high: 162.7 min/day), MPA (low: 12.1 min/day; medium: 26.9 min/day; high: 63.6 min/day), and VPA (low: 0.5 min/day; medium: 1.7 min/day; high: 5.5 min/day).

Reference groups in the joint associations were defined as participants with high sedentary time (top tertile) and low physical activity (bottom tertile). Multivariable models were adjusted for age, sex, education, race and ethnicity, smoking status, alcohol consumption, fruits and vegetables, sleep duration (device-measured), family history of CVD and cancer and mutually-adjusted for intensities of physical activity (e.g., joint associations of sedentary time and VPA were adjusted for LPA and MPA) based on a priori defined directed acyclic graph of previous studies from our group [28]. Of note, we detected no evidence for multicollinearity using VIF tests and the correlation between physical activity intensities were low (LPA vs. MPA: *r* = 0.29; LPA vs. VPA: *r* = 0.06; and MPA vs. VPA: *r* = 0.27).

We performed sensitivity analyses by:

Adding BMI, hypertension, diabetes, and self-rated health status (i.e., collected prior to the accelerometer data) into the main model.

Replacing fruits and vegetables by dietary pattern score [30, 31] into the main model.

Using traditional acceleration magnitude cut-offs of device-measured physical activity [32].

Using Fine and Grey models to account for competing risks for CVD vs. cancer mortality.

All statistical analysis were performed in the Stata version 17.0.

## Results

During a median of 6.9 years of follow-up (628,807 person-years), we documented 1521 deaths, including 388 from CVD. Baseline characteristics of the 73,729 participants by joint sedentary time and MVPA groups are displayed in the Table 1. Compared to participants with high MVPA and low sedentary time, those with low MVPA and high sedentary time were older and more likely men, current smokers, never drinkers, had lower consumption of fruits and vegetables; higher educational attainment, BMI, hypertension and diabetes and family history of CVD and cancer (Table 1). Participants characteristics by LPA, MPA and VPA are displayed in the Supplementary Table S1.

**Table 1:** Baseline characteristics of participants by device-measured sedentary time and moderate to vigorous physical activity, UK Biobank

	High MVPA/ Low ST	High MVPA/ Medium ST	High MVPA/ High ST	Medium MVPA/ Low ST	Medium MVPA/ Medium ST	Medium MVPA/ High ST	Low MVPA/ Low ST	Low MVPA/ Medium ST	Low MVPA/ High ST
	(*n* = 14,546)	(*n* = 7,495)	(*n* = 3,823)	(*n* = 7,656)	(*n* = 9,563)	(*n* = 7,729)	(*n* = 3,457)	(*n* = 7,678)	(*n* = 11,782)
**Sedentary time, median hours/day**	9.6	10.6	11.6	9.5	10.7	12.0	9.6	10.8	12.2
**LPA, median min/day**	140.4	90.8	75.8	123.8	94.7	74.9	94.2	81.9	66.2
**MPA, median min/day**	58.5	49.9	47.7	28.4	26.8	26.1	13.9	12.9	11.1
**VPA, median min/day**	4.2	3.8	3.2	2.0	2.0	1.8	0.9	0.8	0.6
**Sleep duration, median hours/day**	7.5	7.1	6.7	7.7	7.4	6.9	7.9	7.6	7.0
**Age, years**	59.6	60.4	61.2	60.7	61.7	63.0	61.2	63.7	65.2
**Men, %**	38.9	49.2	56.0	37.6	43.5	49.6	33.5	37.1	44.6
**Race and ethnicity, %**									
Asian	1.0	1.2	1.5	1.2	1.0	1.3	1.1	1.1	1.4
Black	0.8	1.0	1.3	0.6	0.8	1.3	0.7	0.7	1.0
Mixed	0.6	0.5	0.9	0.5	0.6	0.8	0.4	0.5	0.6
Other	0.8	0.7	1.1	0.6	0.8	0.1	1.0	0.7	1.1
White	96.8	96.7	95.3	97.0	96.7	95.4	96.7	97.0	95.9
**Educational attainment (years of study), %**									
College or University degree (20)	42.6	48.2	50.4	43.5	45.1	46.1	38.8	43.5	43.5
A levels/AS levels or equivalent (13)	13.8	12.9	13.6	13.8	13.0	13.5	12.7	14.0	13.1
O levels/GSCEs or equivalent (10)	20.9	19.1	17.1	21.1	21.0	19.6	23.2	19.9	20.0
CSEs or equivalent (10)	5.4	4.1	3.5	4.7	4.0	3.2	5.3	3.9	3.0
NVQ or HND or HNC or equivalent (19)	5.2	4.9	4.9	5.1	5.2	5.0	5.8	4.8	5.5
Other	12.1	10.8	10.4	11.9	11.5	12.6	13.8	13.9	15.0
**BMI, kg/m**^**2**^, **mean (sd)**	24.7	25.5	26.2	25.2	25.8	26.5	26.3	26.4	27.5
**Smoking status, %**									
Current cigarette	5.7	6.0	6.2	6.4	6.2	6.8	7.8	7.3	8.4
Former cigarette	33.5	34.2	34.8	33.8	34.7	34.5	34.3	34.8	35.7
Never cigarette	60.8	59.9	59.1	59.8	59.1	58.8	58.0	57.9	55.9
**Alcohol intake**									
Never	2.7	2.4	3.1	2.4	2.5	2.5	3.4	3.0	3.8
Former	2.4	2.3	3.3	2.2	2.2	2.7	2.6	2.6	3.2
Current, below guideline	56.6	55.6	54.2	57.5	57.9	57.8	57.8	59.2	58.6
Current, above guideline	38.3	39.7	39.4	37.9	37.4	37.1	36.2	35.2	34.4
**Fruits and Vegetables, serving/day**	8.4	8.2	8.2	8.2	8.0	7.9	8.1	7.9	7.8
**Family history of CVD**	51.4	52.1	53.2	53.7	53.2	53.8	53.2	55.2	56.7
**Family history of cancer**	24.1	23.6	25.0	24.9	25.1	25.1	23.9	26.1	25.4
**Diabetes, %**	0.4	0.2	0.6	0.5	0.6	0.6	0.5	0.7	1.0
**Hypertension, %**	8.5	10.7	12.5	11.3	12.6	15.9	14.6	15.5	22.0

**Abbreviation**: MVPA: Moderate to vigorous physical activity; MPA: moderate physical activity; VPA: vigorous physical activity; LIPA: light physical activity; BMI: body mass index; CVD: cardiovascular disease. NVQ National Vocational Qualification (NVQ) or HND Higher National Diploma (HND) or HNC Higher National Certificate (HNC) or equivalent = 19 years of education; CSEs Certificate of Secondary education (CSE) or equivalent = 10 years of education; O levels/GSCEs General Certificate of Secondary Education (GCSE) or equivalent = 10 years of education; A levels/AS levels or equivalent = 13 years of education; and College or University degree = 20 years of education. Tertiles were used to categorize low (3.1 to 10.1 h/day), medium (10.2 to 11.3 h/day) and high (≥ 11 h/day) sedentary time. We also used tertiles to categorize MVPA (0 to 21.2 min/day; 21.3 to 40.9 min/day; ≥41 min/day), LPA (0 to 70.2 min/day; 70.3 to 119.2 min/day; ≥119.3 min/day), MPA (0 to 19.1 min/day; 19.2 to 36.8 min/day; ≥36.9 min/day), and VPA (0 to 0.97 min/day; 0.98 to 2.9 min/day; ≥3 min/day)

### Association of sedentary time with all-cause and CVD mortality

Sedentary time was associated with higher risk of all-cause mortality, but not CVD mortality. Compared to low sedentary time (≤ 10.1 h/day), multivariable-adjusted HR for all-cause mortality were 1.06 (95% CI 0.91 to 1.23) for medium (10.2 to 11.3 h/day) and 1.28 (95% CI 1.08 to 1.51) for high (≥ 11.4 h/day) sedentary time. Multivariable-adjusted HR for CVD mortality were 1.16 (95% CI 0.85 to 1.58) for medium sedentary time and 1.10 (95% CI 0.78 to 1.56) for high sedentary time.

### Joint association of sedentary time and intensity-specific physical activity with all-cause mortality

**Fig. 1:**
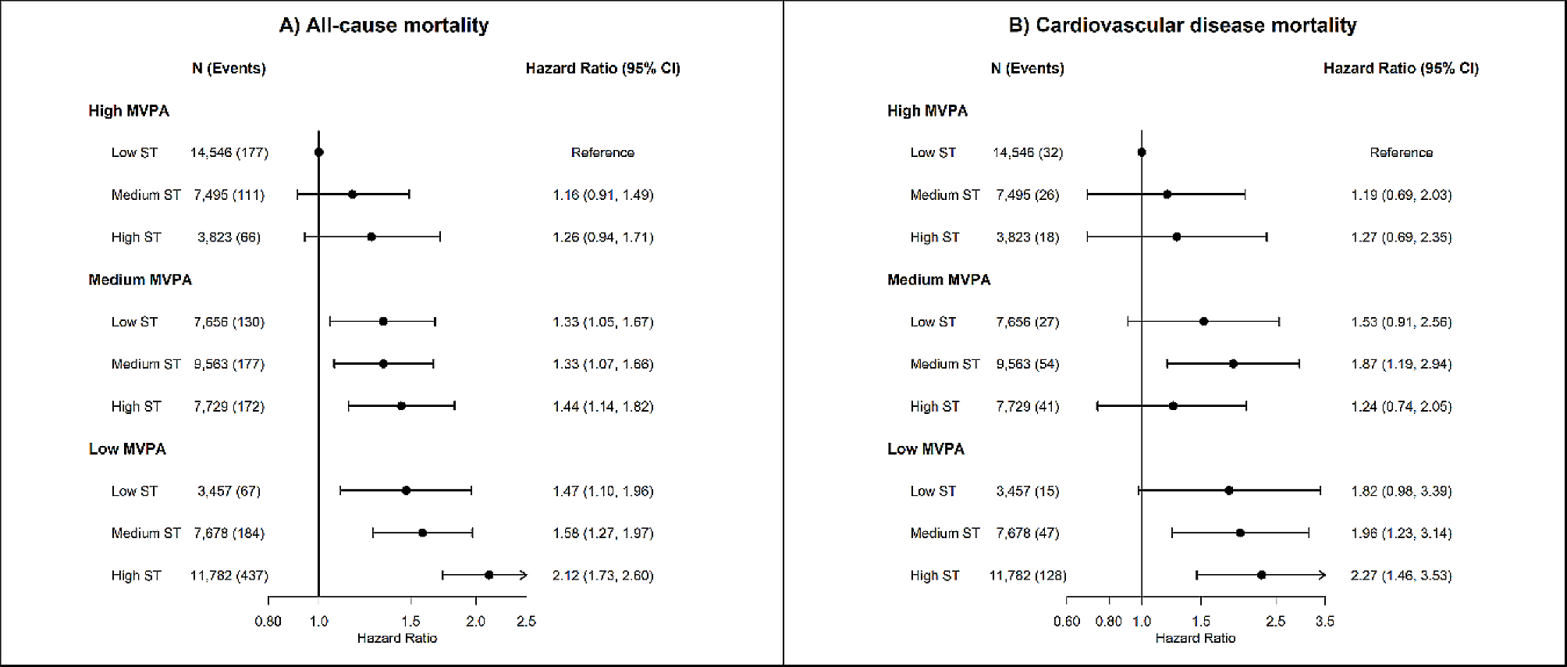
Joint associations of device-measured sedentary time and moderate-to-vigorous physical activity with all-cause and cardiovascular disease mortality. *Footnote*: Tertiles were used to categorize low (3.1 to 10.1 h/day), medium (10.2 to 11.3 h/day) and high (≥ 11.3 h/day) sedentary time; and MVPA (0–21 min/day; 21–41 min/day; >41 min/day). The median value of MVPA time in each tertile were: low: 13.5 min/day; medium: 30.0 min/day; high: 59.3 min/day

In joint association analyses, sedentary time was associated with higher risk of all-cause mortality among participants with low and medium MVPA, but less so among those with high MVPA (Fig. 1). Among participants with high MVPA (a median of 60 min/day) the association between sedentary time and all-cause mortality was attenuated (Fig. 1). Compared to participants with low sedentary time and high MVPA, multivariable-adjusted HR for all-cause mortality were 1.16 (95% CI 0.91 to 1.49) for medium sedentary time and high MVPA, 1.33 (95% CI 1.07 to 1.66) for medium sedentary time and medium MVPA, and 1.58 (95% CI 1.27 to 1.97) for medium sedentary time and low MVPA. Considering the same reference group, multivariable-adjusted HR for all-cause mortality were 1.26 (95% CI 0.94 to 1.71) for high sedentary time and high MVPA, 1.44 (95% CI 1.14 to 1.82) for high sedentary time and medium MVPA, and 2.12 (95% CI 1.73 to 2.60) for high sedentary time and low MVPA.

**Fig. 2:**
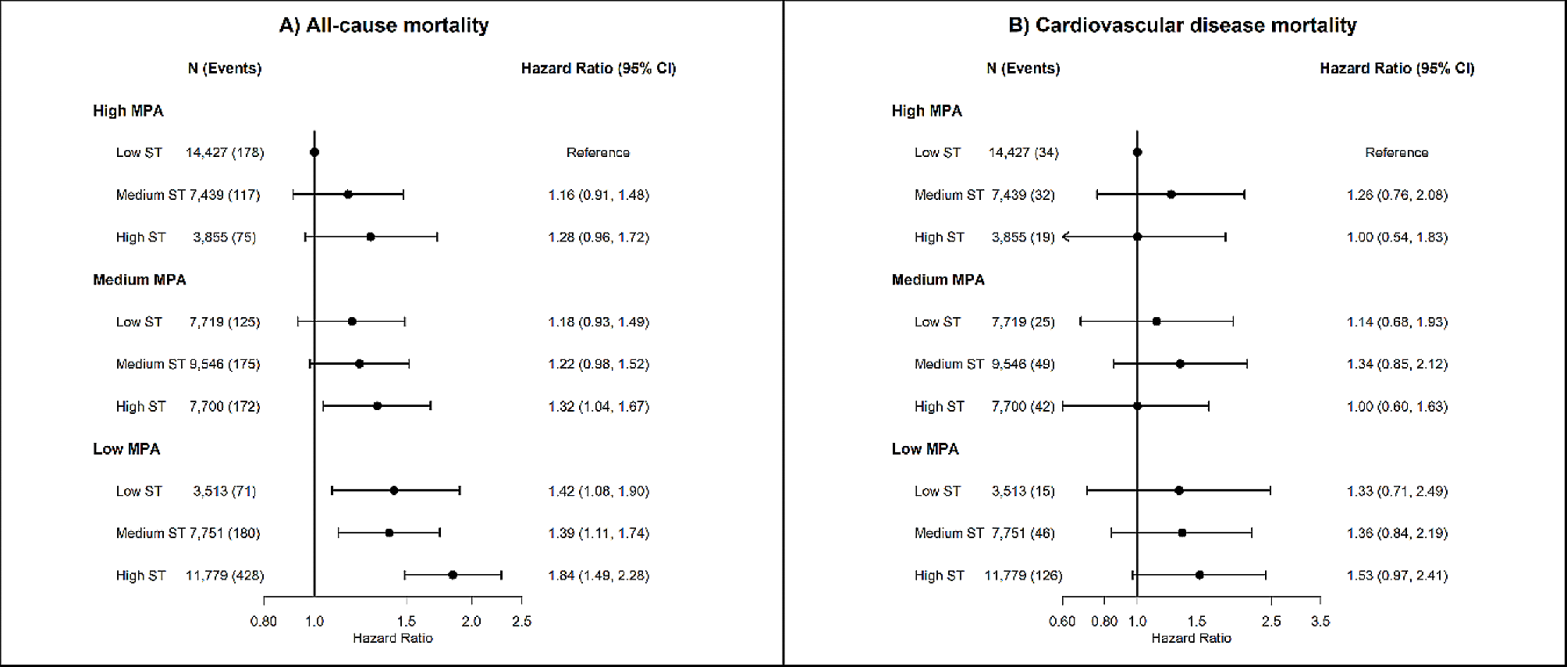
Joint associations of device-measured sedentary time and moderate physical activity with all-cause and cardiovascular disease mortality. *Footnote*: Tertiles were used to categorize low (3.1 to 10.1 h/day), medium (10.2 to 11.3 h/day) and high (≥ 11.3 h/day) sedentary time; and MPA (< 19 min/day; 19–36.8 min/day; >36.8 min/day). The median value of MPA time in each tertile were: low: 54.1 min/day; medium: 90.4 min/day; high: 162.7 min/day

**Fig. 3:**
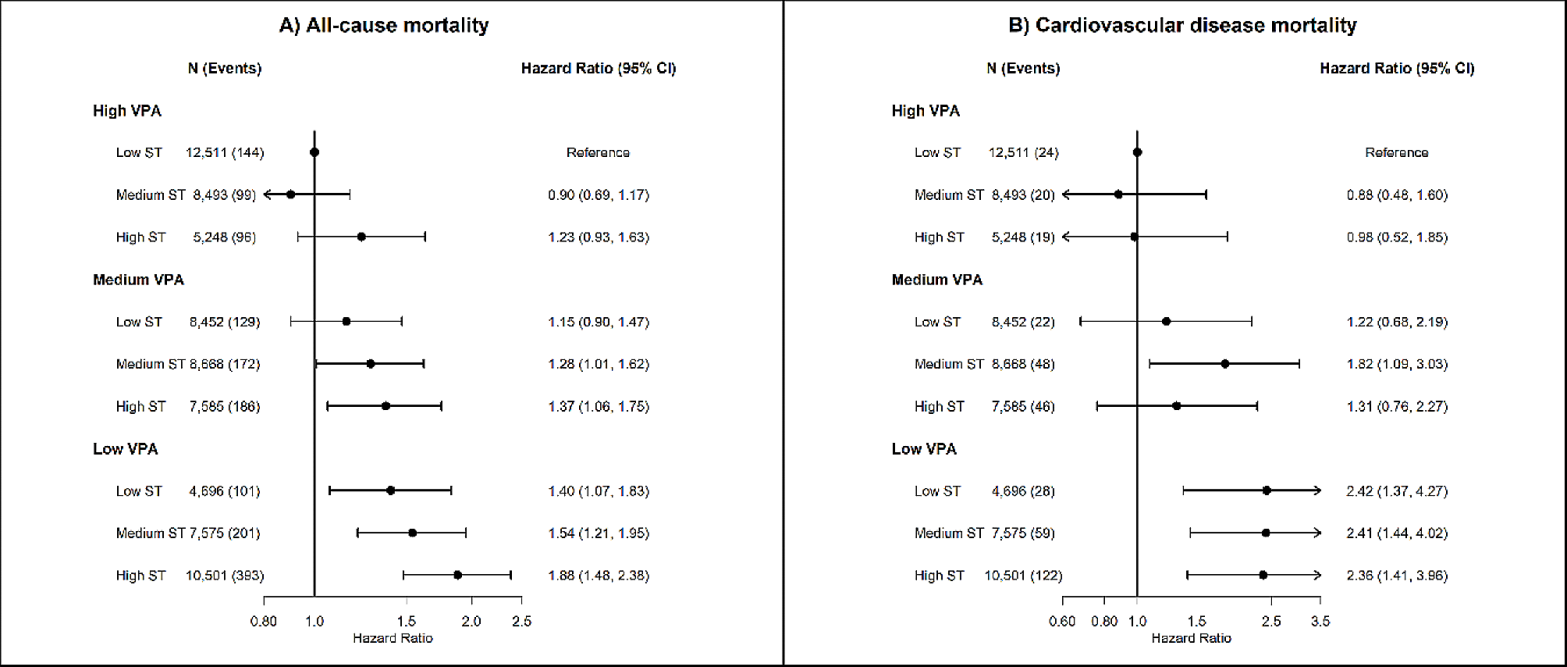
Joint associations of device-measured sedentary time and vigorous physical activity with all-cause and cardiovascular disease mortality. Footnote: Tertiles were used to categorize low (3.1 to 10.1 h/day), medium (10.2 to 11.3 h/day) and high (≥ 11.3 h/day) sedentary time; and VPA (< 1 min/day; 1–3 min/day; >3 min/day). The median value of VPA time in each tertile were: low: 0.5 min/day; medium: 1.7 min/day; high: 5.5 min/day

**Fig. 4:**
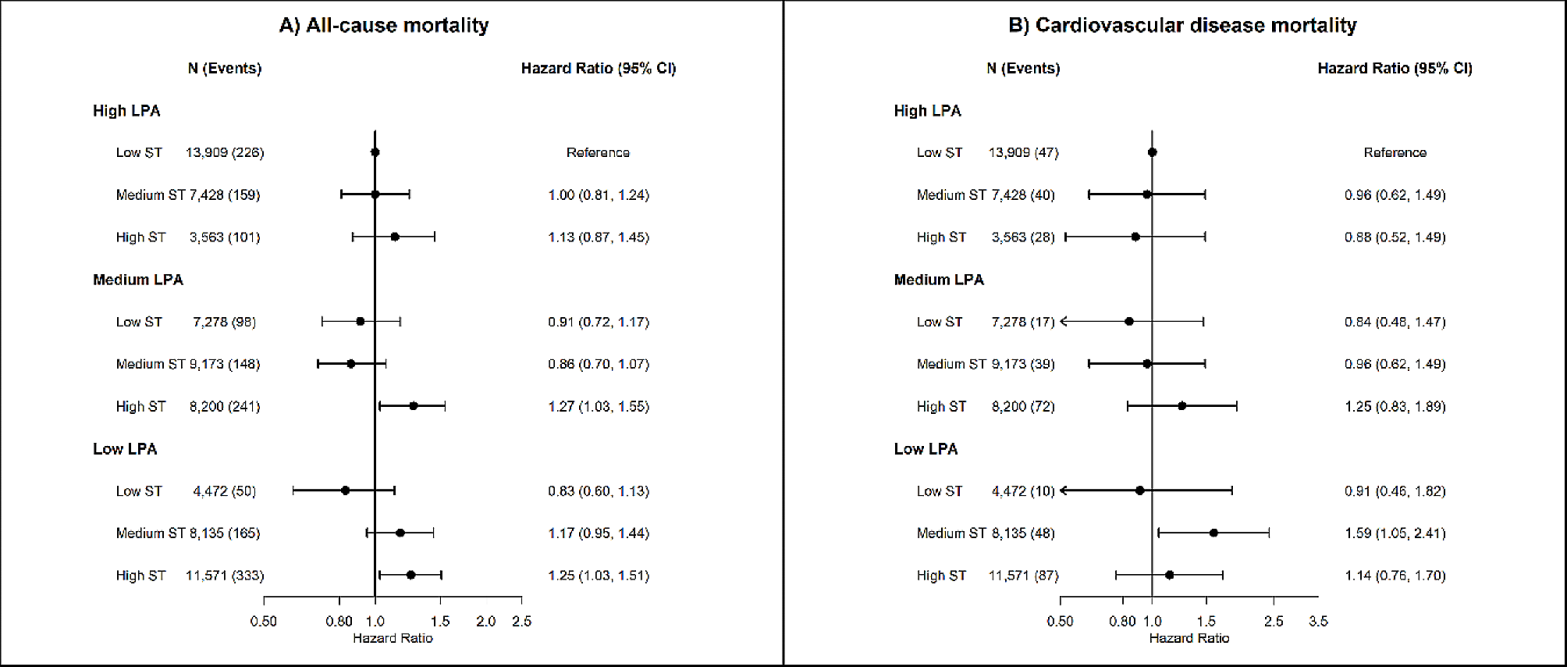
Joint associations of device-measured sedentary time and light physical activity with all-cause and cardiovascular disease mortality. Footnote: Tertiles were used to categorize low (3.1 to 10.1 h/day), medium (10.2 to 11.3 h/day) and high (≥ 11.3 h/day) sedentary time; and LPA (< 70 min/day; 70–119 min/day; >119 min/day). The median value of LPA time in each tertile were: low: low: 54.1 min/day; medium: 90.4 min/day; high: 162.7 min/day

Similar patterns of association were found for joint associations of sedentary time with MPA (Fig. 2) and VPA (Fig. 3), but not with LPA (Fig. 4). Compared to participants with low sedentary time and high MPA, multivariable-adjusted HR for all-cause mortality were 1.28 (95% CI 0.96 to 1.72) for high sedentary time and high MPA, 1.34 (95% CI 1.04 to 1.67) for high sedentary time and medium MPA, and 1.84 (95% CI 1.49 to 2.28) for high sedentary time and low MPA. For LPA, we found an association of high sedentary time with higher risk of all-cause mortality among participants with low (HR 1.25; 95% CI 1.03 to 1.51) and medium (HR 1.27; 95% CI 1.03 to 1.55), but not among participants with high LPA (HR 1.13; 95% CI 0.87 to 1.45).

Overall, at least a median of 6 min/day of VPA, 30 min/day of MVPA, 64 min/day of MPA, or 163 min/day of LPA (mutually adjusted for other intensities) attenuated most of the association between sedentary time and mortality (Fig. 3).

### Joint association of sedentary time and intensity-specific physical activity with cardiovascular disease mortality

We did not observe a clear joint association of sedentary time and intensity-specific physical activity with CVD mortality (Fig. 1). Sedentary time was associated with higher risk of CVD mortality only among participants with low MVPA. Compared to participants with low sedentary time and high MVPA, multivariable-adjusted HR for CVD mortality were 1.96 (95% CI 1.23 to 3.14) for medium sedentary time and low MVPA, and 2.27 (95% CI 1.46 to 3.53) for high sedentary time and low MVPA (Fig. 1). Similar results were observed for joint association of sedentary time and VPA, with consistent positive associations between sedentary time and CVD mortality only among participants with low VPA. Compared to participants with low sedentary time and high VPA, multivariable HR for CVD mortality were 2.42 (95% CI 1.37 to 4.27) for low sedentary time and low VPA, 2.41 (95% CI 1.44 to 4.02) for medium sedentary time and low VPA, and 2.36 (95% CI 1.41 to 3.96) for high sedentary time and low VPA (Fig. 3). We did not observe an association between sedentary time and higher risk of CVD mortality across groups of MPA (Fig. 2) and LPA, with low number of CVD deaths and wide 95% CI estimates (Fig. 3).

### Sensitivity analysis

In the sensitivity analyses, adding BMI, hypertension, diabetes, and self-rated health into the model, replacing fruits and vegetables with diet pattern score, and using traditional acceleration magnitude cut-offs of device-measured physical activity did not materially influence the main results. Fine Grey models to account for competing risks for CVD showed similar results compared to main results (Table S2-6).

## Discussion

In this large prospective cohort study of wearable movement sensors, high sedentary time was associated with higher risk of all-cause mortality, but not CVD mortality. Of note, medium sedentary time was associated with both all-cause and CVD mortality. One possible explanation for this inconsistent finding is the distribution of the amount of movement intensities across the sedentary time groups, which is not adequately captured in the independent association between sedentary time and mortality; highlighting the importance of our study on the joint distribution of sedentary time and intensity-specific physical activity in regards to mortality. Of note, we observed that high sedentary time was consistently associated with all-cause and CVD mortality among participants with low levels of physical activity of any intensity. We also found that physical activity of any intensity attenuated the associations between sedentary time and higher risk of all-cause mortality. A median of at least 6 min/day of VPA, 30 min/day of MVPA, 64 min/day of MPA, or 163 min/day of LPA attenuated the association between sedentary time and mortality. For MPA and VPA, the attenuations were more consistent across the sedentary time groups for all-cause mortality than for CVD mortality. For all-cause mortality, we observed a dose-response relationship with sedentary time across all MPA and VPA groups, with weaker associations as physical activity increased. For CVD, however, we did not observe the same pattern of associations across the joint categories of sedentary time and MPA and VPA, particularly in the medium MPA and VPA groups. Sedentary time was associated with higher risk of CVD mortality only among participants with low levels of MVPA and VPA. High sedentary time was associated with higher risk of all-cause and CVD mortality among participants with low LPA, although LPA results presented wide CI and thus should carefully interpreted.

### Comparison with other studies

A prospective cohort study (45 and Up Study) of Australian adults showed that self-reported sitting time was associated with all-cause mortality among insufficiently active participants (defined as doing < 150 min/week of MVPA, equivalent to approximately 22 min/day), but not among those meeting (150–300 min/week of MVPA) or exceeding the physical activity recommendations [3]. Similarly, a harmonized meta-analysis of self-reported data from more than 1 million adults showed that daily sitting time was not associated with higher risk of all-cause mortality among participants with > 35.5 MET-h per week of activity (i.e., approximately 60 to 75 min of MPA per day) [2]. The self-reported measures of sedentary behavior and physical activity used in previous studies reflect person’s behavior, while accelerometers suffer from different types of measurement error [7, 22]. Lower exposure measurement errors have been associated with greater magnitude of association of sedentary behavior and physical activity with mortality [7, 22]. Self-reported and accelerometer-based measures of activity are related but different constructs (self-reported captures behavioral blocks while accelerometers capture most movement). Questionnaires, for example, usually collect information on selected physical activity domains, mostly leisure-time, and enquire about bouts lasting ≥ 10–15 continuous minutes, whereas accelerometry captures all domains and bout lengths. Accelerometer-based studies, however, also have some limitations and challenges, such as the use of the device in single point in time (24-hrs/day for 7 days), which may lead to regression dilution bias. Hip and wrist accelerometers usually do not assess posture well i.e., definition of sedentary time requires both low energy expenditure and sitting/lying position. Possibly, accelerometers can capture occupational PA less accurately than other PA domains due to their inability to accurately record upper body activity and/or less locomotor activities. In addition, device-measured sedentary time and physical activity present limited contextual information, typically do not capture water-based activities, participants may not wear the accelerometer as instructed and the accuracy of measurements can be influenced by demographic factors. Finally, large-scale epidemiological studies can require significant financial and human resources. Considering all these limitations and challenges, our all-cause mortality results are supported by a harmonized meta-analysis of accelerometer-measured physical activity and sedentary time including over 44 thousand participants [21]. In their accelerometer-based study including nine prospective cohort studies grom four countries (Norway, Sweden, United States of America and United Kingdom), 44,370 adults (69.7% women; mean age 65.8 years and standard deviation 8.6 years) were followed for 4 to 15 years and 3451 deaths occurred. Higher sedentary time was associated with higher risk of all-cause mortality in less active participants, but not among those doing 30 to 40 min/day of movement at MVPA intensity. Our findings add new evidence to guide practitioners and public health by providing specific amounts of intensity-specific physical activity needed to attenuate the association between sedentary time and mortality. Future wearables-based studies from diverse cohorts would benefit from reproducing our joint analyses and expanding to other analytical paradigms, such as CODA, which is a powerful method specifically developed to examine replacement effects of time spent in 24-hour composition components (e.g. sleep, different postures, different physical activity intensities).

### Clinical & public health implications

Our findings underscore that high sedentary time is associated with higher risk of all-cause and CVD mortality, particularly among participants with low physical activity. Moderate and vigorous intensities, in particular, attenuated the association of sedentary time with mortality risk, but larger amounts of LPA may also hold promise. Specifically, we showed that a median of 6 min/day of VPA, 30 min/day of MVPA, 64 min/day of MPA, or 163 min/day of LPA may attenuate the association between high sedentary time (> 11 h/day) and mortality. These movement thresholds could be used to guide quantitative intensity-specific physical activity targets for people who are highly sedentary and wear consumer level monitoring device (e.g., fitness trackers or smartwatches). Our findings may inform clinical practice and personalized prescription for behavioral counselling, as well as and public health guidelines, including future device-based guidelines on physical activity and sedentary time. Considering the high prevalence of prolonged sedentary behaviour and insufficient physical activity in the population, [33] such clinical and public health guidelines are warranted.

### Strengths and limitations

Our study has several strengths, including the use of device-measures of sedentary time and physical activity intensity and the machine learning-based classification scheme. However, there are also several limitations. Device-measures of sedentary time and physical activity intensities were captured only in a single point in time. Repeated-measures of physical activity are less prone to measurement error and show greater magnitudes of association with mortality compared to a single measurement at baseline [7, 22] Although we excluded participants diagnosed with CVD, cancer or respiratory diseases prior to the accelerometry baseline and excluded deaths within the first two years of follow-up, the possibility of reverse causation remains. Second, the large number of sedentary time and physical activity groups compromised the precision of the HR estimates, generating wide 95% CI, particularly in the LPA and CVD mortality analyses. Although there was a median lag of 5.5 years between the UK Biobank baseline when covariates measurements and the accelerometry study, covariates were stable over time, with the exception of medication [8]. Considering this lag-time, it is plausible to assume that covariates were ancestors of exposure, and therefore potential common causes of both exposure and outcomes (aka confounders) [28, 34]. As this is an observational study, although we accounted for several potential confounders, residual confounding may still exist. The UK Biobank 2006–2010 baseline had a 5.5% response rate and accelerometer participants were a subgroup of respondents (e.g., higher education attainment and thus more likely to have sedentary jobs), therefore participants were not representative of the overall UK population of adults aged 40–69. However, recent empirical evidence suggests that poor representativeness does not materially influence the association between physical activity and mortality in the UK Biobank [35].

## Conclusions

High sedentary time was associated with higher risk of all-cause mortality. Physical activity of any intensity attenuated the associations of sedentary time and all-cause mortality: a median of 6 min/day of VPA, 30 min/day of MVPA, 64 min/day of MPA, or 163 min/day of LPA. The attenuations provided by MPA and VPA intensities were more consistent across the sedentary time groups for all-cause mortality than for CVD mortality. For CVD mortality, high sedentary time (≥ 11 h/day) was associated with higher risk of mortality only among participants with low levels of physical activity (54 min/day of LPA; 12 min/day of MPA; and < 1 min/day of VPA).

### Electronic supplementary material

Below is the link to the electronic supplementary material.

Supplementary Material 1

Supplementary Material 2

## Data Availability

The UK Biobank data that support the findings of this study can be accessed by researchers on application (https://www.ukbiobank.ac.uk/register-apply/).
