# Supplementary material for: Device-measured sedentary time and intensity-specific physical activity in relation to all-cause and cardiovascular disease mortality: the UK Biobank cohort study
Source: Int J Behav Nutr Phys Act. 2024 Jul 3;21:68. doi: 10.1186/s12966-024-01615-5 (PMC11223286; doi:10.1186/s12966-024-01615-5)
Supplement: Supplementary file 2 — Supplementary Material 2 [file 12966_2024_1615_MOESM2_ESM.docx]

**Table S1**. Baseline characteristics of participants by device-measured sedentary time and intensity-specific physical activity, UK Biobank

|  | **Sedentary time and light physical activity (LPA)** | | | | | | | | |
| --- | --- | --- | --- | --- | --- | --- | --- | --- | --- |
|  | **High LPA/ Low ST** | **High LPA/**  **Medium ST** | **High LPA/**  **High ST** | **Medium LPA/ Low ST** | **Medium LPA/**  **Medium ST** | **Medium LPA/**  **High ST** | **Low LPA/ Low ST** | **Low LPA/**  **Medium ST** | **Low LPA/**  **High ST** |
|  |  |  |  |  |  |  |  |  |  |
| **Sedentary time, median hours/day** | 9.1 | 10.6 | 12.1 | 9.2 | 10.7 | 12.2 | 9.3 | 10.7 | 12.4 |
| **LPA, median min/day** | 181.4 | 160.7 | 154.2 | 93.2 | 91.9 | 90.3 | 55.3 | 53.8 | 51.1 |
| **MPA, median min/day** | 54.2 | 32.2 | 27.7 | 45.2 | 30.5 | 23.6 | 41.1 | 29.4 | 21.1 |
| **VPA, median min/day** | 4.7 | 3.1 | 2.4 | 5.2 | 3.6 | 2.6 | 5.0 | 3.5 | 2.1 |
| **Sleep duration, median hours/day** | 7.5 | 7.0 | 6.4 | 7.7 | 7.4 | 6.7 | 7.8 | 7.6 | 7.0 |
| **Age, years** | 60.2 | 61.5 | 62.6 | 59.2 | 61.0 | 62.4 | 58.9 | 60.8 | 62.7 |
| **Men, %** | 38.8 | 47.8 | 52.8 | 37.8 | 43.5 | 49.0 | 34.3 | 38.7 | 46.0 |
| **Race and ethnicity, %** |  |  |  |  |  |  |  |  |  |
| Asian | 0.9 | 1 | 1.5 | 1.2 | 0.9 | 1.4 | 1.3 | 1.4 | 1.3 |
| Black | 0.8 | 1.2 | 1.4 | 0.6 | 0.6 | 1.1 | 0.9 | 0.8 | 1.2 |
| Mixed | 0.5 | 0.5 | 0.8 | 0.7 | 0.6 | 0.8 | 0.5 | 0.5 | 0.6 |
| Other | 0.6 | 0.8 | 1 | 0.9 | 0.7 | 1.2 | 1.1 | 0.8 | 1 |
| White | 97.1 | 96.6 | 95.3 | 96.7 | 97.2 | 95.5 | 96.2 | 96.5 | 95.9 |
| **Educational attainment (years of study), %** |  |  |  |  |  |  |  |  |  |
| College or University degree (20) | 42.0 | 44.6 | 44.1 | 42.7 | 46.5 | 45.3 | 42.8 | 45.5 | 46 |
| A levels/AS levels or equivalent (13) | 13.6 | 13.3 | 13.9 | 14.2 | 13 | 13.6 | 13.3 | 13.6 | 12.8 |
| O levels/GSCEs or equivalent (10) | 21.1 | 20.3 | 20 | 20.8 | 19.8 | 19.6 | 22.5 | 20.2 | 19.1 |
| CSEs or equivalent (10) | 5.2 | 3.6 | 3.5 | 5.2 | 4.3 | 3 | 5.2 | 4 | 3.1 |
| NVQ or HND or HNC or equivalent (19) | 5.2 | 5.6 | 5 | 5.4 | 4.9 | 5.4 | 5 | 4.5 | 5.2 |
| Other | 12.8 | 12.7 | 13.5 | 11.8 | 11.5 | 13.1 | 11.2 | 12.2 | 13.7 |
| **BMI, kg/m^2^, mean (sd)** | 25.6 | 26.9 | 27.8 | 25.6 | 26.3 | 27.6 | 25.4 | 26.2 | 27.7 |
| **Smoking status, %** |  |  |  |  |  |  |  |  |  |
| Current cigarette | 6 | 6.4 | 6.9 | 6.2 | 6.6 | 8.2 | 6.9 | 6.4 | 7.2 |
| Former cigarette | 34 | 35.3 | 36 | 34.4 | 34.6 | 35.2 | 31.8 | 33.9 | 34.8 |
| Never cigarette | 60.1 | 58.3 | 57.1 | 59.4 | 58.8 | 56.6 | 61.3 | 59.8 | 58 |
| **Alcohol intake** |  |  |  |  |  |  |  |  |  |
| Never | 2.7 | 2.8 | 3.1 | 2.8 | 2.3 | 3.2 | 2.6 | 2.8 | 3.3 |
| Former | 2.5 | 2.6 | 3.7 | 2.2 | 2.2 | 3.1 | 2.2 | 2.3 | 2.7 |
| Current, below guideline | 57.4 | 56.7 | 56.2 | 56.5 | 57.3 | 56.1 | 56.6 | 58.7 | 59.2 |
| Current, above guideline | 37.4 | 37.8 | 37 | 38.4 | 38.2 | 37.6 | 38.5 | 36.2 | 34.8 |
| **Fruits and Vegetables, serving/day** | 8.4 | 8.1 | 7.9 | 8.3 | 8.1 | 8.0 | 8.2 | 8.0 | 7.9 |
| **Family history of CVD** | 52.8 | 54 | 56.8 | 52.4 | 53.2 | 54.8 | 50.8 | 53.4 | 54.9 |
| **Family history of cancer** | 24.9 | 25.3 | 25 | 23.7 | 25.5 | 24.4 | 23.6 | 24 | 25.9 |
| **Diabetes, %** | 0.5 | 0.6 | 1.0 | 0.5 | 0.4 | 0.6 | 0.2 | 0.5 | 0.8 |
| **Hypertension, %** | 10.7 | 15.0 | 20.3 | 9.7 | 12.2 | 17.4 | 9.0 | 11.9 | 18.5 |
|  | **Sedentary time and moderate physical activity (MPA)** | | | | | | | | |
|  | **High MPA/ Low ST** | **High MPA/**  **Medium ST** | **High MPA/**  **High ST** | **Medium MPA/ Low ST** | **Medium MPA/**  **Medium ST** | **Medium MPA/**  **High ST** | **Low MPA/ Low ST** | **Low MPA/**  **Medium ST** | **Low MPA/**  **High ST** |
|  |  |  |  |  |  |  |  |  |  |
| **Sedentary time, median hours/day** | 8.9 | 10.6 | 12.1 | 9.3 | 10.7 | 12.2 | 9.4 | 10.7 | 12.4 |
| **LPA, median min/day** | 145.1 | 105.4 | 89.3 | 129.2 | 104.7 | 86.4 | 101.7 | 89.1 | 73.9 |
| **MPA, median min/day** | 69.6 | 56.3 | 52.8 | 28.1 | 27.1 | 26.6 | 13.1 | 12.3 | 10.9 |
| **VPA, median min/day** | 6.0 | 4.9 | 3.9 | 3.9 | 3.4 | 2.8 | 2.3 | 1.9 | 1.5 |
| **Sleep duration, median hours/day** | 7.5 | 7.1 | 6.6 | 7.7 | 7.3 | 6.8 | 7.9 | 7.6 | 6.9 |
| **Age, years** | 59.5 | 60.3 | 61.2 | 59.9 | 60.9 | 61.9 | 60.1 | 61.9 | 63.5 |
| **Men, %** | 37.8 | 47.1 | 53.2 | 38.9 | 43.6 | 49.1 | 35.1 | 39.1 | 45.8 |
| **Race and ethnicity, %** |  |  |  |  |  |  |  |  |  |
| Asian | 1.0 | 1.1 | 1.3 | 1.2 | 1.1 | 1.5 | 1.2 | 1.1 | 1.4 |
| Black | 0.8 | 1.1 | 1.3 | 0.7 | 0.8 | 1.4 | 0.7 | 0.7 | 1.0 |
| Mixed | 0.6 | 0.5 | 0.8 | 0.6 | 0.6 | 0.8 | 0.5 | 0.5 | 0.6 |
| Other | 0.8 | 0.7 | 1 | 0.6 | 0.7 | 1.1 | 1 | 0.8 | 1.1 |
| White | 96.8 | 96.6 | 95.6 | 97 | 96.8 | 95.2 | 96.6 | 97 | 96 |
| **Educational attainment (years of study), %** |  |  |  |  |  |  |  |  |  |
| College or University degree (20) | 42.3 | 47.2 | 48.8 | 43.3 | 45.3 | 46 | 40.3 | 44.3 | 44 |
| A levels/AS levels or equivalent (13) | 13.7 | 12.7 | 13.5 | 13.9 | 13.1 | 13.3 | 13.4 | 14.2 | 13.2 |
| O levels/GSCEs or equivalent (10) | 21.1 | 19.5 | 17.9 | 21 | 20.9 | 19.6 | 22.6 | 19.6 | 19.8 |
| CSEs or equivalent (10) | 5.5 | 4.2 | 3.6 | 4.8 | 3.9 | 3.2 | 5.1 | 4 | 2.9 |
| NVQ or HND or HNC or equivalent (19) | 5.1 | 4.9 | 5.1 | 5.3 | 5.4 | 5 | 5.7 | 4.6 | 5.4 |
| Other | 12.4 | 11.5 | 11.1 | 11.7 | 11.5 | 12.8 | 13 | 13.3 | 14.7 |
| **BMI, kg/m^2^, mean (sd)** | 25.2 | 26.2 | 26.9 | 25.8 | 26.3 | 27.2 | 26.8 | 27.0 | 28.2 |
| **Smoking status, %** |  |  |  |  |  |  |  |  |  |
| Current cigarette | 5.7 | 6 | 6.3 | 6.4 | 6.2 | 6.8 | 7.7 | 7.2 | 8.3 |
| Former cigarette | 33.4 | 34.7 | 35 | 34.1 | 34.5 | 34.7 | 33.9 | 34.6 | 35.4 |
| Never cigarette | 60.9 | 59.3 | 58.7 | 59.5 | 59.3 | 58.4 | 58.4 | 58.3 | 56.2 |
| **Alcohol intake** |  |  |  |  |  |  |  |  |  |
| Never | 2.8 | 2.6 | 2.8 | 2.5 | 2.4 | 2.6 | 3.2 | 2.9 | 3.8 |
| Former | 2.4 | 2.3 | 3.3 | 2.1 | 2.2 | 2.7 | 2.6 | 2.6 | 3.2 |
| Current, below guideline | 57 | 56 | 54.7 | 56.9 | 58.1 | 57.8 | 57.5 | 58.5 | 58.4 |
| Current, above guideline | 37.9 | 39.2 | 39.2 | 38.5 | 37.3 | 36.9 | 36.7 | 36 | 34.6 |
| **Fruits and Vegetables, serving/day** | 8.4 | 8.2 | 8.1 | 8.3 | 7.9 | 7.9 | 8.0 | 7.9 | 7.8 |
| **Family history of CVD** | 51.5 | 52.6 | 53.1 | 53.9 | 53.2 | 54.5 | 52.3 | 54.8 | 56.2 |
| **Family history of cancer** | 24.2 | 24 | 25.2 | 24.7 | 25.2 | 24.9 | 23.9 | 25.6 | 25.5 |
| **Diabetes, %** | 0.4 | 0.2 | 0.6 | 0.4 | 0.6 | 0.6 | 0.5 | 0.7 | 1.0 |
| **Hypertension, %** | 8.7 | 11.3 | 13.5 | 11.2 | 12.7 | 16.0 | 13.7 | 14.9 | 21.6 |
|  | **Sedentary time and vigorous physical activity (VPA)** | | | | | | | | |
|  | **High VPA/ Low ST** | **High VPA/**  **Medium ST** | **High VPA/**  **High ST** | **Medium VPA/ Low ST** | **Medium VPA/**  **Medium ST** | **Medium VPA/**  **High ST** | **Low VPA/ Low ST** | **Low VPA/**  **Medium ST** | **Low VPA/**  **High ST** |
|  |  |  |  |  |  |  |  |  |  |
| **Sedentary time, median hours/day** | 8.9 | 10.6 | 12.2 | 9.2 | 10.7 | 12.2 | 9.3 | 10.7 | 12.4 |
| **LPA, median min/day** | 131.3 | 98.6 | 83.9 | 138.5 | 101.0 | 81.8 | 135.4 | 100.5 | 78.1 |
| **MPA, median min/day** | 57.9 | 39.6 | 32.5 | 45.5 | 31.2 | 25.4 | 33.6 | 22.1 | 16.5 |
| **VPA, median min/day** | 8.5 | 7.6 | 6.9 | 1.8 | 1.8 | 1.7 | 0.5 | 0.5 | 0.4 |
| **Sleep duration, median hours/day** | 7.5 | 7.2 | 6.7 | 7.6 | 7.3 | 6.8 | 7.8 | 7.5 | 6.8 |
| **Age, years** | 58.3 | 59.0 | 59.8 | 60.4 | 51.2 | 62.3 | 62.3 | 63.2 | 64.2 |
| **Men, %** | 45.1 | 54.9 | 63.5 | 33.6 | 42.5 | 51.1 | 25.6 | 30.9 | 38.2 |
| **Race and ethnicity, %** |  |  |  |  |  |  |  |  |  |
| Asian | 1.1 | 1.3 | 1.8 | 0.9 | 1.1 | 1.4 | 1.3 | 0.9 | 1.2 |
| Black | 0.9 | 1.1 | 2.0 | 0.7 | 0.7 | 1 | 0.6 | 0.6 | 0.9 |
| Mixed | 0.6 | 0.6 | 0.8 | 0.6 | 0.5 | 0.6 | 0.3 | 0.4 | 0.7 |
| Other | 0.8 | 0.7 | 1.3 | 0.7 | 0.8 | 1 | 0.8 | 0.7 | 1.1 |
| White | 96.6 | 96.2 | 94.2 | 97.1 | 96.9 | 96 | 97 | 97.3 | 96.1 |
| **Educational attainment (years of study), %** |  |  |  |  |  |  |  |  |  |
| College or University degree (20) | 44.2 | 49 | 51.1 | 40.8 | 44.7 | 44.6 | 40.1 | 42.8 | 43.2 |
| A levels/AS levels or equivalent (13) | 13.5 | 13.3 | 13.6 | 14.3 | 13.6 | 13.6 | 13.4 | 13 | 12.9 |
| O levels/GSCEs or equivalent (10) | 20.9 | 18.7 | 17.5 | 21.4 | 20.7 | 19.8 | 22.1 | 21 | 20.2 |
| CSEs or equivalent (10) | 5.3 | 4.4 | 3.4 | 5.4 | 3.6 | 3.2 | 4.7 | 3.9 | 3 |
| NVQ or HND or HNC or equivalent (19) | 5.4 | 5.1 | 5 | 5.2 | 5 | 5.4 | 4.8 | 4.9 | 5.2 |
| Other | 10.8 | 9.5 | 9.5 | 12.9 | 12.5 | 13.4 | 14.9 | 14.4 | 15.5 |
| **BMI, kg/m^2^, mean (sd)** | 24.9 | 25.6 | 26.5 | 25.9 | 26.5 | 27.3 | 26.9 | 27.4 | 28.5 |
| **Smoking status, %** |  |  |  |  |  |  |  |  |  |
| Current cigarette | 5.8 | 5.9 | 5.8 | 6 | 6.4 | 7.7 | 7.5 | 7.2 | 8.2 |
| Former cigarette | 32.9 | 33.7 | 33.4 | 34.5 | 35.5 | 35.8 | 34.4 | 34.4 | 35.5 |
| Never cigarette | 61.3 | 60.4 | 60.8 | 59.5 | 58.1 | 56.5 | 58.1 | 58.4 | 56.3 |
| **Alcohol intake** |  |  |  |  |  |  |  |  |  |
| Never | 2.5 | 2.3 | 2.5 | 2.7 | 2.3 | 2.9 | 3.5 | 3.3 | 3.9 |
| Former | 2.1 | 2.1 | 2.4 | 2.4 | 2.1 | 2.9 | 3 | 3 | 3.4 |
| Current, below guideline | 55.1 | 54.7 | 54.1 | 58.1 | 57.8 | 55.6 | 60.1 | 60.6 | 60.8 |
| Current, above guideline | 40.3 | 40.9 | 41 | 36.8 | 37.8 | 38.6 | 33.4 | 33.1 | 31.9 |
| **Fruits and Vegetables, serving/day** | 8.4 | 8.0 | 7.9 | 8.3 | 8.1 | 7.9 | 8.3 | 8.0 | 7.9 |
| **Family history of CVD** | 50.4 | 50.1 | 51.7 | 52.6 | 53.9 | 53.9 | 56.9 | 56.8 | 57.8 |
| **Family history of cancer** | 23.3 | 23.4 | 23.7 | 25.3 | 25.9 | 25.3 | 25.2 | 25.7 | 26.0 |
| **Diabetes, %** | 0.4 | 0.4 | 0.4 | 0.4 | 0.5 | 0.7 | 0.4 | 0.6 | 1.0 |
| **Hypertension, %** | 7. | 9.0 | 11.4 | 11.6 | 12.8 | 16.9 | 15.8 | 17.5 | 23.0 |

**Abbreviation:** MVPA: Moderate to vigorous physical activity; MPA: moderate physical activity; VPA: vigorous physical activity; LIPA: light physical activity; BMI: body mass index; CVD: cardiovascular disease. NVQ National Vocational Qualification (NVQ) or HND Higher National Diploma (HND) or HNC Higher National Certificate (HNC) or equivalent = 19 years of education; CSEs Certificate of Secondary education (CSE) or equivalent = 10 years of education; O levels/GSCEs General Certificate of Secondary Education (GCSE) or equivalent = 10 years of education; A levels/AS levels or equivalent = 13 years of education; and College or University degree = 20 years of education. Tertiles were used to categorize low (3.1 to 10.1 hours/day), medium (10.2 to 11.3 hours/day) and high (≥11 hours/day) sedentary time. We also used tertiles to categorize MVPA (0 to 21.2 min/day; 21.3 to 40.9 min/day; ≥41 min/day), LPA (0 to 70.2 min/day; 70.3 to 119.2 min/day; ≥119.3 min/day), MPA (0 to 19.1 min/day; 19.2 to 36.8 min/day; ≥36.9 min/day), and VPA (0 to 0.97 min/day; 0.98 to 2.9 min/day; ≥3 min/day).

**Supplemental Text 1:** Physical activity classification6

Physical activity was classified using a previously validated Random Forest (RF) activity classifier.^1^ RF is an ensemble of multiple decision trees. Each tree is learned on a bootstrap sample of training data and each node in the tree is split using the best among a randomly selected set of acceleration features. The decisions from each tree are aggregated and a final model prediction is based on majority vote. The RF model requires very little pre-processing of the data, as the features do not need to be normalized. Additionally, the model is resistant to over fitting the training data because each tree within the forest is independently grown to maximum depth using a randomly selected subset of features.

The classifier categorized physical activity in 10 second windows into 1 of 4 activity classes: sedentary, standing utilitarian movements (ironing a shirt, washing dishes), walking activities (gardening, active commuting, mopping floors), running/high energetic activities (active playing with children). These activities were then assigned to 1 of 4 activity intensities: sedentary, light, moderate, and vigorous. Walking activities were classified as light (<100mg), moderate (≥100mg) and vigorous (≥400mg) intensity. The diagram below depicts how activity intensity was classified Differentiation from sleep^2^ and non-wear^3^ was identified using the change in tilt angle and acceleration standard deviation. Monitors were calibrated^4^ and corrected for orientation^5^ using previously published methods.


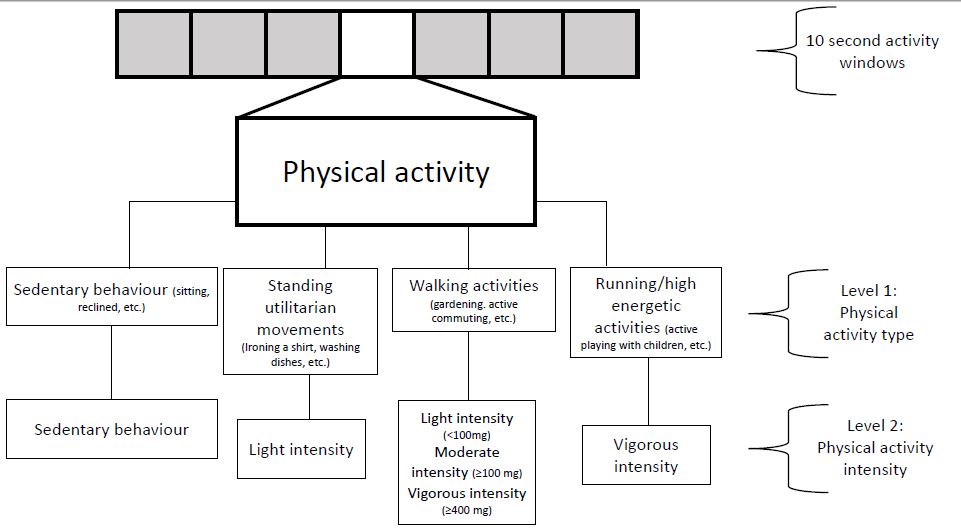
**Physical activity type and intensity diagram**

Activities in an independent sample of 98 participants (Age = 56.4 ± 15.7; 53.1% female) from the US^6^ (University of California Irvine Center for Machine Learning and Intelligent Systems *Physical Activity Monitoring for Aging People* study [published data], accessible at https://archive.ics.uci.edu/ml/datasets) and Australia^7^ (University of Queensland *Where and When at Work* study [published data], and University of Sydney *Intermittent Lifestyle Physical Activity* Study [unpublished data]) providing 103,607 activity samples from structured and free-living activities (17,267 minutes) were used to assess robustness and generalizability of the classifier. For free-living activities participant-worn or researcher-held Go-Pro video-recordings were used to attain ground-truth physical activity. Video files were imported into the Noldus Observer XT software for continuous direct observation coding. A two-stage direct observation scheme was implemented in which the participant’s movement behaviour was coded for activity type and then activity intensity based on Compendium of Physical Activities^8^. The direct observation system generated a vector of date-time stamps corresponding to the start and finish of each movement event, which were used to assign the activity codes to the corresponding time segments of the accelerometer data. Interobserver reliability was assessed by dual coding. The intraclass correlation coefficient for coding activities was 0.912 (0.866-0.942).

Performance was further evaluated in a separate sample of 151 adults (age range 18-91 years, 65.6% female) recruited from the UK^9^ (University of Oxford *Capture 24* study [published data], accessible at https://ora.ox.ac.uk/objects/uuid:99d7c092-d865-4a19-b096-cc16440cd001). Participants in this dataset wore body cameras that provided pictures every 20 seconds to annotate ground-truth free-living activity labels. The picture-based activity coding scheme has been previously described^9^. A total of 172,360 activity samples (28,727 minutes) were provided by participants.

Classifier performance in the three datasets is provided below:

Intensity classification performance

|  | Sensitivity | Specificity | Precision | F-score | Overall Accuracy | Weighted Kappa | Overall F-score |
| --- | --- | --- | --- | --- | --- | --- | --- |
| Sedentary | 86.5 | 93.7 | 90.5 | 88.5 |  |  |  |
| Light | 71.2 | 89.4 | 55.8 | 62.6 |  |  |  |
| Moderate | 85.4 | 96.6 | 92.7 | 88.9 |  |  |  |
| Vigorous | 95.4 | 99.4 | 94.6 | 95.0 |  |  |  |
|  |  |  |  |  | 84.6 | 0.78 | 83.8 |

Confusion matrix

|  | Sedentary | Light | Moderate | Vigorous |
| --- | --- | --- | --- | --- |
| Sedentary | **36,904** | 5,232 | 508 | 2 |
| Light | 3,120 | **11,712** | 1,612 | 17 |
| Moderate | 502 | 4,016 | **29,528** | 526 |
| Vigorous | 226 | 17 | 214 | **9,470** |
| Rows= ground truth; columns=predictions; bold=correct labels; numbers represent each 10-second window; Derived from the US and Australian datasets | | | | |

**
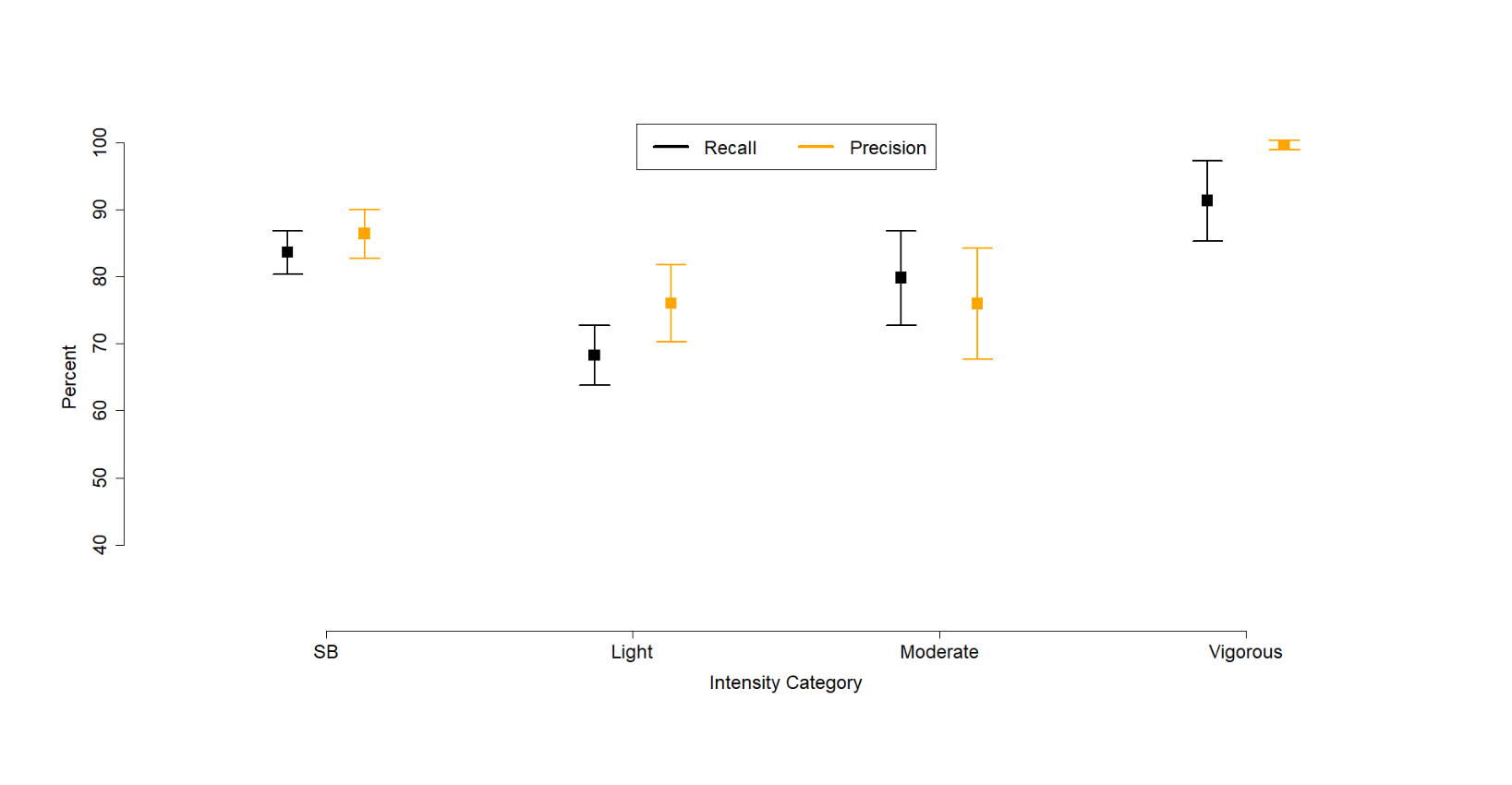
Participant-level specific recall and precision in the UK sample**

**Table S2**: Joint associations of device-measured sedentary time and specific-intensity physical activity with all-cause and cause-specific mortality, UK Biobank

|  |  | **All-cause mortality** | |  | **CVD mortality** | |
| --- | --- | --- | --- | --- | --- | --- |
|  | **n** | **Deaths** | **Multivariable model** |  | **Deaths** | **Multivariable model** |
|  |  |  | **HR (95% CI)** |  |  | **HR (95% CI)** |
| **Joint sedentary time and MVPA*** |  |  |  |  |  |  |
| Low sedentary time and high MVPA | 14,546 | 177 | 1.00 [Reference] |  | 32 | 1.00 [Reference] |
| Medium sedentary time and high MVPA | 7,495 | 111 | 1.16 (0.91-1.49) |  | 26 | 1.19 (0.69-2.03) |
| High sedentary time and high MVPA | 3,823 | 66 | 1.26 (0.94-1.71) |  | 18 | 1.27 (0.69-2.35) |
| Low sedentary time and medium MVPA | 7,656 | 130 | 1.33 (1.05-1.67) |  | 27 | 1.53 (0.91-2.56) |
| Medium sedentary time and medium MVPA | 9,563 | 177 | 1.33 (1.07-1.66) |  | 54 | 1.87 (1.19-2.94) |
| High sedentary time and medium MVPA | 7,729 | 172 | 1.44 (1.14-1.82) |  | 41 | 1.24 (0.74-2.05) |
| Low sedentary time and low MVPA | 3,457 | 67 | 1.47 (1.10-1.96) |  | 15 | 1.82 (0.98-3.39) |
| Medium sedentary time and low MVPA | 7,678 | 184 | 1.58 (1.27-1.97) |  | 47 | 1.96 (1.23-3.14) |
| High sedentary time and low MVPA | 11,782 | 437 | 2.12 (1.73-2.6) |  | 128 | 2.27 (1.46-3.53) |
| **Joint sedentary time and MPA**** |  |  |  |  |  |  |
| Low sedentary time and high MPA | 14,427 | 178 | 1.00 [Reference] |  | 34 | 1.00 [Reference] |
| Medium sedentary time and high MPA | 7,439 | 117 | 1.16 (0.91-1.48) |  | 32 | 1.26 (0.76-2.08) |
| High sedentary time and high MPA | 3,855 | 75 | 1.28 (0.96-1.72) |  | 19 | 1.00 (0.54-1.83) |
| Low sedentary time and medium MPA | 7,719 | 125 | 1.18 (0.93-1.49) |  | 25 | 1.14 (0.68-1.93) |
| Medium sedentary time and medium MPA | 9,546 | 175 | 1.22 (0.98-1.52) |  | 49 | 1.34 (0.85-2.12) |
| High sedentary time and medium MPA | 7,700 | 172 | 1.32 (1.04-1.67) |  | 42 | 1.00 (0.60-1.63) |
| Low sedentary time and low MPA | 3,513 | 71 | 1.42 (1.08-1.90) |  | 15 | 1.33 (0.71-2.49) |
| Medium sedentary time and low MPA | 7,751 | 180 | 1.39 (1.11-1.74) |  | 46 | 1.36 (0.84-2.19) |
| High sedentary time and low MPA | 11,779 | 428 | 1.84 (1.49-2.28) |  | 126 | 1.53 (0.97-2.41) |
| **Joint sedentary time and VPA#** |  |  |  |  |  |  |
| Low sedentary time and high VPA | 12,511 | 144 | 1.00 [Reference] |  | 24 | 1.00 [Reference] |
| Medium sedentary time and high VPA | 8,493 | 99 | 0.90 (0.69-1.17) |  | 20 | 0.88 (0.48-1.60) |
| High sedentary time and high VPA | 5,248 | 96 | 1.23 (0.93-1.63) |  | 19 | 0.98 (0.52-1.85) |
| Low sedentary time and medium VPA | 8,452 | 129 | 1.15 (0.90-1.47) |  | 22 | 1.22 (0.68-2.19) |
| Medium sedentary time and medium VPA | 8,668 | 172 | 1.28 (1.01-1.62) |  | 48 | 1.82 (1.09-3.03) |
| High sedentary time and medium VPA | 7,585 | 186 | 1.37 (1.06-1.75) |  | 46 | 1.31 (0.76-2.27) |
| Low sedentary time and low VPA | 4,696 | 101 | 1.40 (1.07-1.83) |  | 28 | 2.42 (1.37-4.27) |
| Medium sedentary time and low VPA | 7,575 | 201 | 1.54 (1.21-1.95) |  | 59 | 2.41 (1.44-4.02) |
| High sedentary time and low VPA | 10,501 | 393 | 1.88 (1.48-2.38) |  | 122 | 2.36 (1.41-3.96) |
| **Joint sedentary time and LPA$** |  |  |  |  |  |  |
| Low sedentary time and high LPA | 13,909 | 226 | 1.00 [Reference] |  | 47 | 1.00 [Reference] |
| Medium sedentary time and high LPA | 7,428 | 159 | 1.00 (0.81-1.24) |  | 40 | 0.96 (0.62-1.49) |
| High sedentary time and high LPA | 3,563 | 101 | 1.13 (0.87-1.45) |  | 28 | 0.88 (0.52-1.49) |
| Low sedentary time and medium LPA | 7,278 | 98 | 0.91 (0.72-1.17) |  | 17 | 0.84 (0.48-1.47) |
| Medium sedentary time and medium LPA | 9,173 | 148 | 0.86 (0.70-1.07) |  | 39 | 0.96 (0.62-1.49) |
| High sedentary time and medium LPA | 8,200 | 241 | 1.27 (1.03-1.55) |  | 72 | 1.25 (0.83-1.89) |
| Low sedentary time and low LPA | 4,472 | 50 | 0.83 (0.60-1.13) |  | 10 | 0.91 (0.46-1.82) |
| Medium sedentary time and low LPA | 8,135 | 165 | 1.17 (0.95-1.44) |  | 48 | 1.59 (1.05-2.41) |
| High sedentary time and low LPA | 11,571 | 333 | 1.25 (1.03-1.51) |  | 87 | 1.14 (0.76-1.70) |

Approximate tertiles were used to categorize low, medium and high sedentary time (3-10 hours/day; 10-11 hours/day; >11 hours/day); and actual tertiles were used to categorise MVPA (0-21 min/day; 21-41 min/day; >41 min/day), LIPA (<70 min/day; 70-119 min/day; >119 min/day), MPA (<19 min/day; 19-36.8 min/day; >36.8 min/day), and VPA (<1 min/day; 1-3 min/day; >3 min/day). The median value of physical activity in each tertile were: MVPA (low: 13.5 min/day; medium: 30.0 min/day; high: 59.3 min/day), LPA (low: 54.1 min/day; medium: 90.4 min/day; high: 162.7 min/day), MPA (low: 12.1 min/day; medium: 26.9 min/day; high: 63.6 min/day), and VPA (low: 0.5 min/day; medium: 1.7 min/day; high: 5.5 min/day).

Multivariable model: adjusted for age, sex, education, race/ethnicity, smoking status, alcohol consumption, fruits and vegetables, sleep duration, family history of CVD (for ACM and CVD mortality) and family history of cancer (for ACM and cancer mortality); plus, exclusion of participants with CVD, cancer and COPD at baseline and less than 2 years of follow-up.

* Additionally adjusted for light physical activity

# Additionally adjusted for light and moderate physical activity

$ Additionally adjusted for moderate to vigorous physical activity

** Additionally adjusted for light and vigorous physical activity

**Table S3: Sensitivity analysis:** Independent and joint associations of device-measured sitting time and **moderate to vigorous physical activity** with all-cause and cardiovascular disease mortality, UK Biobank

|  | **Participants** | **Deaths** | **Age- and sex-adjusted model** |  | **Multivariable mode 1** |  | **Multivariable model 2** |  | **Multivariable Model 3** |  | **Traditional cut-offs  (MV1)** |  | **Fine and Grey model** |
| --- | --- | --- | --- | --- | --- | --- | --- | --- | --- | --- | --- | --- | --- |
|  |  |  | **HR (95% CI)** |  | **HR (95% CI)** |  | **HR (95% CI)** |  | **HR (95% CI)** |  | **HR (95% CI)** |  |  |
| **All-Cause Mortality** |  |  |  |  |  |  |  |  |  |  |  |  |  |
| Low ST and high MVPA | 14,546 | 177 | 1.00 [Reference] |  | 1.00 [Reference] |  | 1.00 [Reference] |  | 1.00 [Reference] |  | 1.00 [Reference] |  | NA |
| Medium ST and high MVPA | 7,495 | 111 | 1.13 (0.89 to 1.43) |  | 1.16 (0.91 to 1.49) |  | 1.12 (0.88 to 1.44) |  | 1.17 (0.91 to 1.50) |  | 0.93 (0.72 to 1.21) |  | NA |
| High ST and high MVPA | 3,823 | 66 | 1.21 (0.91 to 1.61) |  | 1.26 (0.94 to 1.71) |  | 1.19 (0.88 to 1.61) |  | 1.29 (0.95 to 1.74) |  | 1.27 (0.95 to 1.71) |  | NA |
| Low ST and medium MVPA | 7,656 | 130 | 1.28 (1.02 to 1.60) |  | 1.33 (1.05 to 1.67) |  | 1.27 (1.01 to 1.60) |  | 1.34 (1.07 to 1.70) |  | 1.12 (0.89 to 1.42) |  | NA |
| Medium ST and medium MVPA | 9,563 | 177 | 1.28 (1.04 to 1.58) |  | 1.33 (1.07 to 1.66) |  | 1.25 (1.01 to 1.56) |  | 1.34 (1.08 to 1.67) |  | 1.16 (0.95 to 1.41) |  | NA |
| High ST and medium MVPA | 7,729 | 172 | 1.42 (1.15 to 1.76) |  | 1.44 (1.14 to 1.82) |  | 1.32 (1.05 to 1.67) |  | 1.46 (1.16 to 1.85) |  | 1.13 (0.92 to 1.40) |  | NA |
| Low ST and low MVPA | 3,457 | 67 | 1.41 (1.07 to 1.87) |  | 1.47 (1.10 to 1.96) |  | 1.34 (1.00 to 1.78) |  | 1.47 (1.10 to 1.96) |  | 1.28 (0.96 to 1.72) |  | NA |
| Medium ST and low MVPA | 7,678 | 184 | 1.51 (1.23 to 1.86) |  | 1.58 (1.27 to 1.97) |  | 1.42 (1.14 to 1.77) |  | 1.60 (1.28 to 2.00) |  | 1.50 (1.24 to 1.82) |  | NA |
| High ST and low MVPA | 11,782 | 437 | 2.08 (1.74 to 2.48) |  | 2.12 (1.73 to 2.6) |  | 1.80 (1.46 to 2.23) |  | 2.15 (1.75 to 2.65) |  | 1.70 (1.43 to 2.02) |  | NA |
|  |  |  | P interaction = 0.3128 |  |  |  |  |  |  |  |  |  |  |
| **Cardiovascular disease mortality** |  |  |  |  |  |  |  |  |  |  |  |  |  |
| Low ST and high MVPA | 14,546 | 32 | 1.00 [Reference] |  | 1.00 [Reference] |  | 1.00 [Reference] |  | 1.00 [Reference] |  | 1.00 [Reference] |  | 1.00 [Reference] |
| Medium ST and high MVPA | 7,495 | 26 | 1.40 (0.83 to 2.34) |  | 1.19 (0.69 to 2.03) |  | 1.11 (0.65 to 1.9) |  | 1.24 (0.72 to 2.13) |  | 0.77 (0.43 to 1.37) |  | 1.19 (0.69 to 2.03) |
| High ST and high MVPA | 3,823 | 18 | 1.69 (0.95 to 3.01) |  | 1.27 (0.69 to 2.35) |  | 1.11 (0.6 to 2.05) |  | 1.34 (0.72 to 2.47) |  | 0.73 (0.34 to 1.56) |  | 1.27 (0.68 to 2.40) |
| Low ST and medium MVPA | 7,656 | 27 | 1.44 (0.86 to 2.40) |  | 1.53 (0.91 to 2.56) |  | 1.4 (0.83 to 2.35) |  | 1.59 (0.94 to 2.67) |  | 0.96 (0.56 to 1.63) |  | 1.52 (0.91 to 2.55) |
| Medium ST and medium MVPA | 9,563 | 54 | 2.04 (1.32 to 3.17) |  | 1.87 (1.19 to 2.94) |  | 1.66 (1.06 to 2.61) |  | 1.92 (1.22 to 3.04) |  | 1.37 (0.92 to 2.03) |  | 1.87 (1.18 to 2.94) |
| High ST and medium MVPA | 7,729 | 41 | 1.73 (1.09 to 2.75) |  | 1.24 (0.74 to 2.05) |  | 1.03 (0.62 to 1.71) |  | 1.28 (0.76 to 2.14) |  | 1.34 (0.89 to 2.02) |  | 1.23 (0.74 to 2.06) |
| Low ST and low MVPA | 3,457 | 15 | 1.72 (0.93 to 3.19) |  | 1.82 (0.98 to 3.39) |  | 1.51 (0.81 to 2.81) |  | 1.77 (0.94 to 3.36) |  | 1.62 (0.92 to 2.86) |  | 1.82 (0.97 to 3.39) |
| Medium ST and low MVPA | 7,678 | 47 | 2.04 (1.30 to 3.21) |  | 1.96 (1.23 to 3.14) |  | 1.56 (0.97 to 2.5) |  | 2.02 (1.25 to 3.24) |  | 1.47 (0.98 to 2.19) |  | 1.95 (1.23 to 3.11) |
| High ST and low MVPA | 11,782 | 128 | 3.09 (2.09 to 4.57) |  | 2.27 (1.46 to 3.53) |  | 1.57 (1 to 2.47) |  | 2.4 (1.54 to 3.76) |  | 1.92 (1.36 to 2.71) |  | 2.25 (1.44 to 3.52) |
|  |  |  | P interaction = 0.2846 |  |  |  |  |  |  |  |  |  |  |
| Multivariable model 1: adjusted for age, sex, education, race/ethnicity, smoking status, alcohol consumption, fruits and vegetables, sleep duration, family history of CVD (for ACM and CVD mortality) and family history of cancer (for ACM and cancer mortality) and light physical activity; plus, exclusion of participants with CVD, cancer and COPD at baseline and less than 2 years of follow-up. | | | | | | | | | | | | | |
| Multivariable model 2: model 1 plus BMI, hypertension and diabetes and self-rated health | | | | | | | | | | | | | |
| Multivariable model 3: model 1 (except for fruits and vegetables) plus diet pattern score | | | | | | | | | | | | | |

**Table S4: Sensitivity analysis:** Independent and joint associations of device-measured sedentary time and **moderate physical activity** with all-cause and cardiovascular disease mortality, UK Biobank

|  | **Participants** | **Deaths** | **Age- and sex-adjusted model** |  | **Multivariable mode 1** |  | **Multivariable model 2** |  | **Multivariable model 3** |  | **Traditional Cut-offs MV1** |  | **Fine and Grey model** |
| --- | --- | --- | --- | --- | --- | --- | --- | --- | --- | --- | --- | --- | --- |
|  |  |  | **HR (95% CI)** |  | **HR (95% CI)** |  | **HR (95% CI)** |  | **HR (95% CI)** |  | **HR (95% CI)** |  |  |
| **ALL-CAUSE MORTALITY** |  |  |  |  |  |  |  |  |  |  |  |  |  |
| Low ST and high MPA | 14247 | 178 | 1.00 [Reference] |  | 1.00 [Reference] |  | 1.00 [Reference] |  | 1.00 [Reference] |  | 1.00 [Reference] |  | 1.00 [Reference] |
| Medium ST and high MPA | 7439 | 117 | 1.17 (0.92 to 1.47) |  | 1.16 (0.91 to 1.48) |  | 1.13 (0.89 to 1.45) |  | 1.16 (0.90 to 1.48) |  | 0.95 (0.74 to 1.23) |  | NA |
| High ST and high MPA | 3855 | 75 | 1.33 (1.01 to 1.74) |  | 1.28 (0.96 to 1.72) |  | 1.22 (0.91 to 1.63) |  | 1.30 (0.97 to 1.75) |  | 1.24 (0.92 to 1.66) |  | NA |
| Low ST and medium MPA | 7719 | 125 | 1.22 (0.97 to 1.54) |  | 1.18 (0.93 to 1.49) |  | 1.16 (0.92 to 1.47) |  | 1.19 (0.94 to 1.51) |  | 1.03 (0.82 to 1.31) |  | NA |
| Medium ST and medium MPA | 9546 | 175 | 1.27 (1.03 to 1.56) |  | 1.22 (0.98 to 1.52) |  | 1.18 (0.95 to 1.47) |  | 1.23 (0.99 to 1.54) |  | 1.10 (0.90 to 1.33) |  | NA |
| High ST and medium MPA | 7700 | 172 | 1.42 (1.15 to 1.75) |  | 1.32 (1.04 to 1.67) |  | 1.25 (0.98 to 1.58) |  | 1.33 (1.05 to 1.69) |  | 1.08 (0.88 to 1.34) |  | NA |
| Low ST and low MPA | 3513 | 71 | 1.52 (1.15 to 2.00) |  | 1.42 (1.07 to 1.90) |  | 1.35 (1.02 to 1.80) |  | 1.42 (1.07 to 1.90) |  | 1.16 (0.86 to 1.55) |  | NA |
| Medium ST and low MPA | 7751 | 180 | 1.49 (1.21 to 1.83) |  | 1.39 (1.11 to 1.74) |  | 1.30 (1.04 to 1.64) |  | 1.40 (1.11 to 1.76) |  | 1.32 (1.09 to 1.61) |  | NA |
| High ST and low MPA | 11779 | 428 | 2.04 (1.71 to 2.44) |  | 1.84 (1.49 to 2.28) |  | 1.64 (1.32 to 2.04) |  | 1.86 (1.50 to 2.31) |  | 1.50 (1.25 to 1.79) |  | NA |
|  |  |  | P interaction = 0.4869 |  |  |  |  |  |  |  |  |  |  |
| **CARDIOVASCULAR DISEASE MORTALITY** |  |  |  |  |  |  |  |  |  |  |  |  |  |
| Low ST and high MPA | 14247 | 34 | 1.00 [Reference] |  | 1.00 [Reference] |  | 1.00 [Reference] |  | 1.00 [Reference] |  | 1.00 [Reference] |  | 1.00 [Reference] |
| Medium ST and high MPA | 7439 | 32 | 1.59 (0.98 to 2.58) |  | 1.26 (0.76 to 2.08) |  | 1.20 (0.72 to 1.98) |  | 1.31 (0.79 to 2.17) |  | 0.71 (0.39 to 1.30) |  | 1.26 (0.76 to 2.07) |
| High ST and high MPA | 3855 | 19 | 1.63 (0.93 to 2.86) |  | 1.00 (0.54 to 1.83) |  | 0.90 (0.49 to 1.65) |  | 1.04 (0.56 to 1.92) |  | 0.81 (0.39 to 1.66) |  | 1.00 (0.53 to 1.88) |
| Low ST and medium MPA | 7719 | 25 | 1.25 (0.74 to 2.09) |  | 1.14 (0.68 to 1.93) |  | 1.10 (0.65 to 1.86) |  | 1.18 (0.69 to 1.99) |  | 0.92 (0.54 to 1.57) |  | 1.14 (0.68 to 1.92) |
| Medium ST and medium MPA | 9546 | 49 | 1.76 (1.14 to 2.73) |  | 1.34 (0.85 to 2.12) |  | 1.26 (0.80 to 1.99) |  | 1.36 (0.86 to 2.17) |  | 1.34 (0.90 to 1.99) |  | 1.34 (0.84 to 2.12) |
| High ST and medium MPA | 7700 | 42 | 1.68 (1.06 to 2.64) |  | 0.99 (0.60 to 1.63) |  | 0.88 (0.53 to 1.46) |  | 1.01 (0.61 to 1.69) |  | 1.20 (0.79 to 1.83) |  | 0.98 (0.59 to 1.63) |
| Low ST and low MPA | 3513 | 15 | 1.65 (0.9 to 3.04) |  | 1.33 (0.71 to 2.49) |  | 1.22 (0.65 to 2.27) |  | 1.29 (0.68 to 2.45) |  | 1.46 (0.82 to 2.59) |  | 1.33 (0.71 to 2.51) |
| Medium ST and low MPA | 7751 | 46 | 1.89 (1.21 to 2.95) |  | 1.36 (0.84 to 2.19) |  | 1.20 (0.74 to 1.94) |  | 1.38 (0.85 to 2.25) |  | 1.29 (0.86 to 1.96) |  | 1.36 (0.84 to 2.20) |
| High ST and low MPA | 11779 | 126 | 2.88 (1.96 to 4.21) |  | 1.53 (0.97 to 2.41) |  | 1.19 (0.75 to 1.89) |  | 1.60 (1.01 to 2.53) |  | 1.71 (1.19 to 2.46) |  | 1.52 (0.95 to 2.43) |
|  |  |  | P interaction = 0.4377 |  |  |  |  |  |  |  |  |  |  |
| Multivariable model 1: adjusted for age, sex, education, race/ethnicity, smoking status, alcohol consumption, fruits and vegetables, sleep duration, family history of CVD (for ACM and CVD mortality) and family history of cancer (ACM and cancer mortality); plus, exclusion of participants with CVD, cancer and COPD at baseline and less than 2 years of follow-up. | | | | | | | | | | | | | |
| Multivariable model 2: model 1 plus BMI, hypertension and diabetes and self-rated health | | | | | | | | | | | | | |
| Multivariable model 3: model 1 (except for fruits and vegetables) plus diet pattern score | | | | | | | | | | | | | |

**Table S5: Sensitivity analysis:** Independent and joint associations of device-measured sedentary time and **vigorous physical activity** with all-cause and cardiovascular disease mortality, UK Biobank

|  | **Participants** | **Deaths** | **Age- and sex-adjusted model** |  | **Multivariable mode 1** |  | **Multivariable model 2** |  | **Multivariable model 3** |  | **Traditional Cut-offs MV1** |  | **Fine and Grey model** |
| --- | --- | --- | --- | --- | --- | --- | --- | --- | --- | --- | --- | --- | --- |
|  |  |  | **HR (95% CI)** |  | **HR (95% CI)** |  | **HR (95% CI)** |  | **HR (95% CI)** |  | **HR (95% CI)** |  |  |
| **ALL-CAUSE MORTALITY** |  |  |  |  |  |  |  |  |  |  |  |  |  |
| Low ST and high VPA | 12,511 | 144 | 1.00 [Reference] |  | 1.00 [Reference] |  | 1.00 [Reference] |  | 1.00 [Reference] |  | 1.00 [Reference] |  | 1.00 [Reference] |
| Medium ST and high VPA | 8,493 | 99 | 0.91 (0.7 to 1.18) |  | 0.90 (0.69 to 1.17) |  | 0.88 (0.68 to 1.15) |  | 0.92 (0.71 to 1.21) |  | 1.08 (0.83 to 1.41) |  | NA |
| High ST and high VPA | 5,248 | 96 | 1.31 (1.01 to 1.69) |  | 1.23 (0.93 to 1.63) |  | 1.19 (0.90 to 1.58) |  | 1.26 (0.95 to 1.67) |  | 1.27 (0.95 to 1.68) |  | NA |
| Low ST and medium VPA | 8,452 | 129 | 1.16 (0.91 to 1.47) |  | 1.15 (0.90 to 1.47) |  | 1.11 (0.87 to 1.42) |  | 1.18 (0.93 to 1.52) |  | 1.32 (1.04 to 1.68) |  | NA |
| Medium ST and medium VPA | 8,668 | 172 | 1.36 (1.09 to 1.7) |  | 1.28 (1.01 to 1.62) |  | 1.21 (0.96 to 1.54) |  | 1.30 (1.02 to 1.65) |  | 1.26 (0.98 to 1.62) |  | NA |
| High ST and medium VPA | 7,585 | 186 | 1.51 (1.21 to 1.88) |  | 1.37 (1.06 to 1.75) |  | 1.26 (0.98 to 1.61) |  | 1.41 (1.09 to 1.81) |  | 1.41 (1.10 to 1.82) |  | NA |
| Low ST and low VPA | 4,696 | 101 | 1.45 (1.12 to 1.88) |  | 1.40 (1.07 to 1.83) |  | 1.28 (0.98 to 1.68) |  | 1.44 (1.10 to 1.88) |  | 1.52 (1.17 to 1.97) |  | NA |
| Medium ST and low VPA | 7,575 | 201 | 1.66 (1.33 to 2.06) |  | 1.54 (1.21 to 1.95) |  | 1.39 (1.09 to 1.77) |  | 1.58 (1.24 to 2.00) |  | 1.88 (1.48 to 2.39) |  | NA |
| High ST and low VPA | 10,501 | 393 | 2.11 (1.73 to 2.56) |  | 1.88 (1.48 to 2.38) |  | 1.62 (1.27 to 2.06) |  | 1.92 (1.51 to 2.43) |  | 2.09 (1.66 to 2.63) |  | NA |
|  |  |  | P interaction = 0.5563 |  |  |  |  |  |  |  |  |  |  |
| **CARDIOVASCULAR DISEASE MORTALITY** |  |  |  |  |  |  |  |  |  |  |  |  |  |
| Low ST and high VPA | 12,511 | 24 | 1.00 [Reference] |  | 1.00 [Reference] |  | 1.00 [Reference] |  | 1.00 [Reference] |  | 1.00 [Reference] |  | 1.00 [Reference] |
| Medium ST and high VPA | 8,493 | 20 | 1.05 (0.58 to 1.91) |  | 0.88 (0.48 to 1.60) |  | 0.86 (0.47 to 1.57) |  | 0.96 (0.52 to 1.78) |  | 1.46 (0.79 to 2.71) |  | 0.88 (0.48 to 1.6) |
| High ST and high VPA | 5,248 | 19 | 1.43 (0.79 to 2.62) |  | 0.98 (0.52 to 1.85) |  | 0.92 (0.49 to 1.73) |  | 1.09 (0.57 to 2.08) |  | 2.13 (1.16 to 3.91) |  | 0.98 (0.51 to 1.88) |
| Low ST and medium VPA | 8,452 | 22 | 1.22 (0.68 to 2.18) |  | 1.22 (0.68 to 2.19) |  | 1.13 (0.63 to 2.02) |  | 1.35 (0.74 to 2.44) |  | 2.01 (1.16 to 3.48) |  | 1.22 (0.68 to 2.19) |
| Medium ST and medium VPA | 8,668 | 48 | 2.22 (1.36 to 3.62) |  | 1.82 (1.09 to 3.03) |  | 1.63 (0.97 to 2.72) |  | 1.97 (1.16 to 3.35) |  | 1.88 (1.06 to 3.34) |  | 1.81 (1.07 to 3.06) |
| High ST and medium VPA | 7,585 | 46 | 2.1 (1.28 to 3.45) |  | 1.31 (0.76 to 2.27) |  | 1.1 (0.63 to 1.91) |  | 1.46 (0.83 to 2.57) |  | 1.85 (1.04 to 3.28) |  | 1.31 (0.75 to 2.29) |
| Low ST and low VPA | 4,696 | 28 | 2.54 (1.47 to 4.4) |  | 2.42 (1.37 to 4.27) |  | 2.05 (1.16 to 3.62) |  | 2.67 (1.50 to 4.78) |  | 2.67 (1.50 to 4.74) |  | 2.42 (1.37 to 4.27) |
| Medium ST and low VPA | 7,575 | 59 | 2.97 (1.84 to 4.79) |  | 2.41 (1.44 to 4.02) |  | 1.96 (1.17 to 3.29) |  | 2.62 (1.55 to 4.45) |  | 2.98 (1.72 to 5.17) |  | 2.4 (1.43 to 4.03) |
| High ST and low VPA | 10,501 | 122 | 3.82 (2.45 to 5.95) |  | 2.36 (1.41 to 3.96) |  | 1.72 (1.02 to 2.92) |  | 2.61 (1.53 to 4.44) |  | 3.66 (2.14 to 6.23) |  | 2.35 (1.39 to 3.95) |
|  |  |  | P interaction = 0.5766 |  |  |  |  |  |  |  |  |  |  |
| Multivariable model 1: adjusted for age, sex, education, race/ethnicity, smoking status, alcohol consumption, fruits and vegetables, sleep duration, family history of CVD (for ACM and CVD mortality) and family history of cancer (ACM and cancer mortality); plus, exclusion of participants with CVD, cancer and COPD at baseline and less than 2 years of follow-up. | | | | | | | | | | | | | |
| Multivariable model 2: model 1 plus BMI, hypertension and diabetes and self-rated health | | | | | | | | | | | | | |
| Multivariable model 3: model 1 (except for fruits and vegetables) plus diet pattern score | | | | | | | | | | | | | |

**Table S6**: **Sensitivity analysis**: Independent and joint associations of device-measured sedentary time and **light physical activity** with all-cause and cardiovascular disease mortality, UK Biobank

|  | **Participants** | **Deaths** | **Age- and sex-adjusted model** |  | **Multivariable model 1** |  | **Multivariable model 2** |  | **Multivariable model 3** |  | **Traditional Cut-offs MV1** |  | **Fine and Grey model** |  |
| --- | --- | --- | --- | --- | --- | --- | --- | --- | --- | --- | --- | --- | --- | --- |
|  |  |  | **HR (95% CI)** |  | **HR (95% CI)** |  | **HR (95% CI)** |  | **HR (95% CI)** |  | **HR (95% CI)** |  |  |  |
| **ALL-CAUSE MORTALITY** |  |  |  |  |  |  |  |  |  |  |  |  |  |  |
| Low ST and high LPA | 13,909 | 226 | 1.00 [Reference] |  | 1.00 [Reference] |  | 1.00 [Reference] |  | 1.00 [Reference] |  | 1.15 (0.92 to 1.43) |  | NA |  |
| Medium ST and high LPA | 7,428 | 159 | 1.12 (0.92 to 1.38) |  | 1.00 (0.81 to 1.24) |  | 0.98 (0.79 to 1.21) |  | 0.99 (0.80 to 1.23) |  | 1.20 (0.95 to 1.51) |  | NA |  |
| High ST and high LPA | 3,563 | 101 | 1.34 (1.06 to 1.69) |  | 1.13 (0.87 to 1.45) |  | 1.05 (0.81 to 1.36) |  | 1.14 (0.88 to 1.47) |  | 0.96 (0.75 to 1.22) |  | NA |  |
| Low ST and medium LPA | 7,278 | 98 | 0.97 (0.77 to 1.23) |  | 0.91 (0.72 to 1.17) |  | 0.92 (0.72 to 1.17) |  | 0.89 (0.70 to 1.14) |  | 0.86 (0.68 to 1.10) |  | NA |  |
| Medium ST and medium LPA | 9,173 | 148 | 0.95 (0.77 to 1.17) |  | 0.86 (0.70 to 1.07) |  | 0.86 (0.69 to 1.06) |  | 0.86 (0.69 to 1.07) |  | 1.38 (1.11 to 1.72) |  | NA |  |
| High ST and medium LPA | 8,200 | 241 | 1.51 (1.26 to 1.81) |  | 1.27 (1.03 to 1.55) |  | 1.21 (0.99 to 1.49) |  | 1.25 (1.02 to 1.53) |  | 1.06 (0.82 to 1.36) |  | NA |  |
| Low ST and low LPA | 4,472 | 50 | 0.88 (0.65 to 1.20) |  | 0.83 (0.60 to 1.13) |  | 0.84 (0.62 to 1.15) |  | 0.83 (0.60 to 1.13) |  | 1.34 (1.07 to 1.68) |  | NA |  |
| Medium ST and low LPA | 8,135 | 165 | 1.33 (1.08 to 1.62) |  | 1.17 (0.95 to 1.44) |  | 1.17 (0.95 to 1.45) |  | 1.17 (0.95 to 1.44) |  | 1.33 (1.07 to 1.65) |  | NA |  |
| High ST and low LPA | 11,571 | 333 | 1.51 (1.28 to 1.79) |  | 1.25 (1.03 to 1.51) |  | 1.18 (0.97 to 1.43) |  | 1.25 (1.03 to 1.52) |  | 1.15 (0.92 to 1.43) |  | NA |  |
|  |  |  | P interaction = 0.0846 |  |  |  |  |  |  |  |  |  |  |  |
| **CARDIOVASCULAR DISEASE MORTALITY** |  |  |  |  |  |  |  |  |  |  |  |  |  |  |
| Low ST and high LPA | 13,909 | 47 | 1.00 [Reference] |  | 1.00 [Reference] |  | 1.00 [Reference] |  | 1.00 [Reference] |  | 1.00 [Reference] |  | 1.00 [Reference] |  |
| Medium ST and high LPA | 7,428 | 40 | 1.28 (0.84 to 1.96) |  | 0.96 (0.62 to 1.49) |  | 0.92 (0.59 to 1.42) |  | 0.98 (0.63 to 1.53) |  | 1.48 (0.92 to 2.37) |  | 0.96 (0.61 to 1.49) |  |
| High ST and high LPA | 3,563 | 28 | 1.64 (1.02 to 2.61) |  | 0.88 (0.52 to 1.49) |  | 0.76 (0.45 to 1.29) |  | 0.92 (0.54 to 1.55) |  | 1.38 (0.84 to 2.27) |  | 0.88 (0.52 to 1.50) |  |
| Low ST and medium LPA | 7,278 | 17 | 0.84 (0.48 to 1.46) |  | 0.84 (0.48 to 1.47) |  | 0.85 (0.49 to 1.48) |  | 0.81 (0.46 to 1.43) |  | 1.33 (0.81 to 2.18) |  | 0.84 (0.48 to 1.48) |  |
| Medium ST and medium LPA | 9,173 | 39 | 1.17 (0.77 to 1.79) |  | 0.96 (0.62 to 1.49) |  | 0.96 (0.62 to 1.49) |  | 0.96 (0.62 to 1.49) |  | 0.81 (0.47 to 1.39) |  | 0.96 (0.62 to 1.49) |  |
| High ST and medium LPA | 8,200 | 72 | 2.04 (1.41 to 2.95) |  | 1.25 (0.83 to 1.89) |  | 1.15 (0.76 to 1.73) |  | 1.25 (0.83 to 1.90) |  | 1.91 (1.21 to 3.03) |  | 1.24 (0.83 to 1.86) |  |
| Low ST and low LPA | 4,472 | 10 | 0.90 (0.45 to 1.78) |  | 0.91 (0.46 to 1.82) |  | 0.96 (0.48 to 1.90) |  | 0.93 (0.47 to 1.85) |  | 1.09 (0.62 to 1.91) |  | 0.91 (0.46 to 1.80) |  |
| Medium ST and low LPA | 8,135 | 48 | 1.88 (1.26 to 2.81) |  | 1.59 (1.05 to 2.41) |  | 1.62 (1.07 to 2.46) |  | 1.59 (1.04 to 2.43) |  | 1.69 (1.04 to 2.74) |  | 1.58 (1.04 to 2.40) |  |
| High ST and low LPA | 11,571 | 87 | 1.81 (1.27 to 2.59) |  | 1.14 (0.76 to 1.70) |  | 1.01 (0.68 to 1.51) |  | 1.18 (0.79 to 1.76) |  | 1.70 (1.07 to 2.68) |  | 1.13 (0.76 to 1.68) |  |
|  |  |  | P interaction = 0.0673 |  |  |  |  |  |  |  |  |  |  |  |
| Multivariable model 1: adjusted for age, sex, education, race/ethnicity, smoking status, alcohol consumption, fruits and vegetables, sleep duration, family history of CVD (ACM and CVD mortality) and family history of cancer (for ACM and cancer mortality); plus, exclusion of participants with CVD, cancer and COPD at baseline and less than 2 years of follow-up. | | | | | | | | | | | | | | |
| Multivariable model 2: model 1 plus BMI, hypertension and diabetes and self-rated health | | | | | | | | | | | | | | |
| Multivariable model 3: model 1 (except for fruits and vegetables) plus diet pattern score | | | | | | | | | | | | | | |
